# Voltage-gated potassium channel proteins and stereoselective *S*-nitroso-l-cysteine signaling

**DOI:** 10.1172/jci.insight.134174

**Published:** 2020-09-17

**Authors:** Benjamin Gaston, Laura Smith, Jürgen Bosch, James Seckler, Diana Kunze, Janna Kiselar, Nadzeya Marozkina, Craig A. Hodges, Patrick Wintrobe, Kellen McGee, Tatiana S. Morozkina, Spencer T. Burton, Tristan Lewis, Timothy Strassmaier, Paulina Getsy, James N. Bates, Stephen J. Lewis

**Affiliations:** 1 Riley Hospital for Children, Indianapolis, Indiana, USA.; 2 Department of Pediatric Pulmonology,; 3 Department of Physiology and Biophysics,; 4 MetroHealth System, and; 5 Department of Proteomics and Bioinformatics, Case Western Reserve University, Cleveland, Ohio, USA.; 6 Department of Pharmaceutical Sciences, University of Maryland, Baltimore, Maryland, USA.; 7 Belarussian State Medical University, Minsk, Belarus.; 8 Nanion Inc., Newark, New Jersey, USA.; 9 Department of Anesthesia, University of Iowa, Iowa City, Iowa, USA.; 10 Department of Pharmacology, Case Western Reserve University, Cleveland, Ohio, USA.

**Keywords:** Cell Biology, Respiration

## Abstract

*S*-nitroso-l-cysteine (L-CSNO) behaves as a ligand. Its soluble guanylate cyclase–independent (sGC-independent) effects are stereoselective — that is, not recapitulated by *S*-nitroso-d-cysteine (D-CSNO) — and are inhibited by chemical congeners. However, candidate L-CSNO receptors have not been identified. Here, we have used 2 complementary affinity chromatography assays — followed by unbiased proteomic analysis — to identify voltage-gated K^+^ channel (Kv) proteins as binding partners for L-CSNO. Stereoselective L-CSNO–Kv interaction was confirmed structurally and functionally using surface plasmon resonance spectroscopy; hydrogen deuterium exchange; and, in Kv1.1/Kv1.2/Kvβ2-overexpressing cells, patch clamp assays. Remarkably, these sGC-independent L-CSNO effects did not involve *S*-nitrosylation of Kv proteins. In isolated rat and mouse respiratory control (petrosyl) ganglia, L-CSNO stereoselectively inhibited Kv channel function. Genetic ablation of Kv1.1 prevented this effect. In intact animals, L-CSNO injection at the level of the carotid body dramatically and stereoselectively increased minute ventilation while having no effect on blood pressure; this effect was inhibited by the L-CSNO congener *S*-methyl-l-cysteine. Kv proteins are physiologically relevant targets of endogenous L-CSNO. This may be a signaling pathway of broad relevance.

## Abstract 2

Two complementary affinity chromatography assays, followed by unbiased proteomic analysis, identified voltage-gated K^+^ channel (Kv) proteins as binding partners for *S*-nitroso-l-cysteine (L-CSNO).

## Introduction

*S*-nitroso-l-cysteine (L-CSNO) is a labile small molecule with bioactivities distinct from those of either l-cysteine or NO. It can be produced in mammals by breakdown of *S*-nitroso-glutathione (GSNO) (1, 2), which is formed by NO synthases (2–5), hemoglobin (Hb) (1, 6–8) and other metalloproteins (2, 9). Enzymes converting GSNO to L-CSNO are involved in regulating several physiological pathways (1, 2, 10, 11). Effects of L-CSNO are not recapitulated by its isomer, *S*-nitroso-d-cysteine (D-CSNO), and are generally opposite those of NO (1, 12–17). Stereoselective, ligand-like neuronal, endothelial, muscular, and immune effects have been described for endogenous L-CSNO (12–19). Specifically, L-CSNO bioactivities reported to date are independent of the NO/soluble guanylate cyclase (sGC) pathway (1, 2, 14, 16, 17). Moreover, specific inhibiters of L-CSNO bioactivities have been developed that are L-CSNO congeners, designed stereoselectively to inhibit the interaction of L-CSNO with target proteins (20). However, a receptor protein has not been identified. We therefore performed an unbiased proteomic analysis of the L-CSNO interactome to identify candidate binding partners that could underlie its ligand-like behavior.

## Results

**Figure 1:**
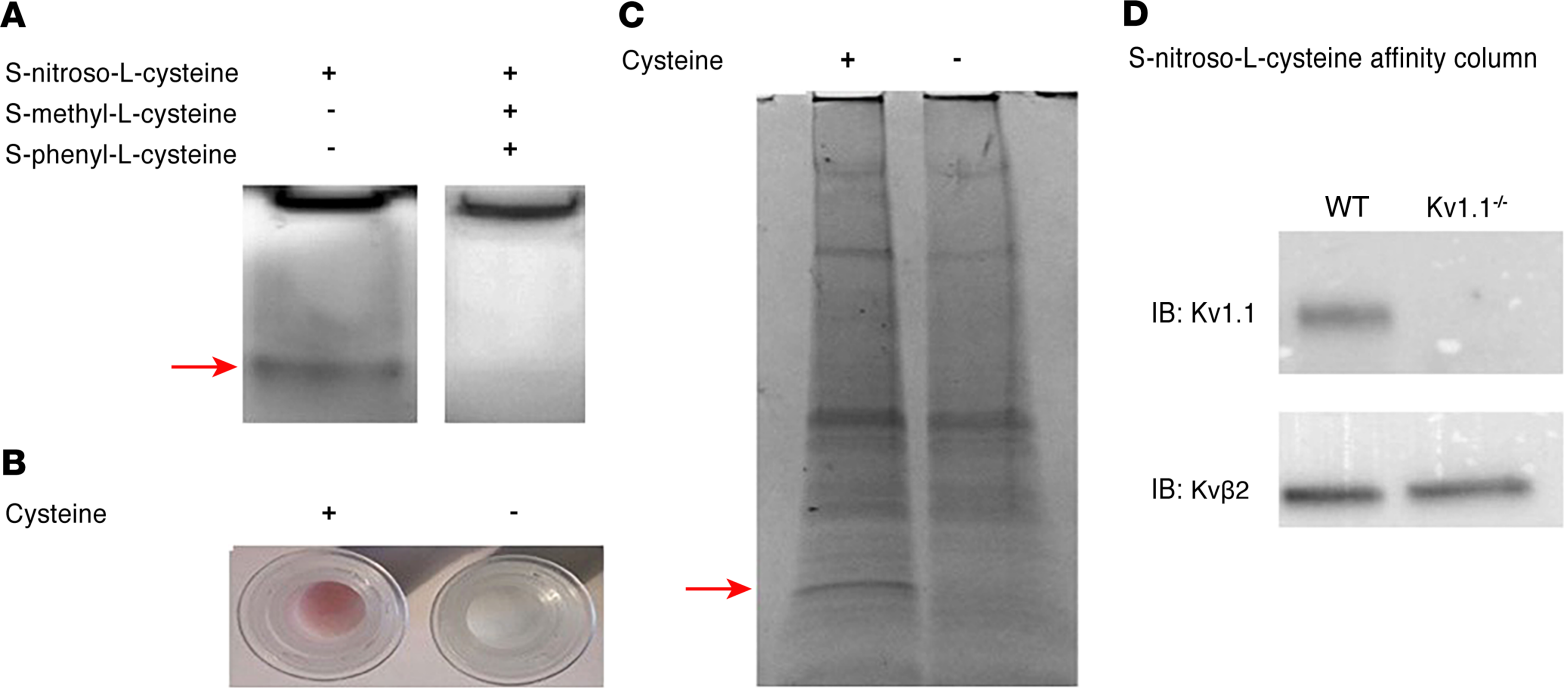
Identification of voltage-gated K^+^ channel proteins as binding partners for L-CSNO. (**A**) Method 1. Murine cortex membrane proteins (50 μg/ lane; 2 sets of experiments using 1 C57BL/6 WT mouse) underwent native PAGE, then were incubated (30 minutes; dark; 27°C) with L-CSNO (50 μM) with or without 100 μM each L-CSMe and L-CSφ. Rinsed gels were developed with 40 μM DAF2 (30 minutes; dark; 27°C), and fluorescing bands (arrow) analyzed by LC-MS (compared with the control lane). Note that, because this is native PAGE, proteins were not separated before electrophoresis. (**B**) Method 2. Cysteine (100 mM) coupled to AminoLink Plus resin (4 hours, 27°C) or resin alone was incubated with EtONO (10 minutes; dark; 27°C). Pink color demonstrates *S*-nitrosothiol formation on the column (37). (**C**) Membrane proteins as in **A** (different animal) were loaded on columns (**B**) (30 min; dark; 27°C), washed, then eluted with Laemmli buffer followed by 0.1 M glycine, pH 3.5. Eluate underwent SDS-PAGE, and bands (including that shown by the arrow, 20 kDa) were analyzed by LC-MS in comparison with the control lane. Both methods identified several Kv channel proteins (see Supplemental Tables 2 and 3). Note that, because native PAGE was used (proteins were not separated before electrophoresis) in A, and a broad region of discordance was excised in **C**, multiple molecular weight proteins were evident by LC-MS. (**D**) Proteins as in **A** from WT and Kv1.1^–/–^ mice (2 sets of experiments using WT and C57BL/6 background mice) were loaded on and eluted from columns as in **B** and **C**, and immunoblotted for Kv1.1 and Kvβ2.

To identify L-CSNO–interacting proteins, we exploited evidence that L-CSNO effects are inhibited by *S*-methyl-l-cysteine (L-CSMe) and *S*-phenyl-cysteine (L-CSφ) (20). In our first method (Method 1), proteins underwent native PAGE. Gels were treated with L-CSNO with or without L-CSMe and L-CSφ and developed with diaminofluorescein-2 (DAF2) (21). Bands fluorescing in the absence of L-CSMe/L-CSφ were compared with control (Figure 1A) by liquid chromatography–mass spectrometry (LC-MS). In Method 2, the cysteine amine was added to beads as a Schiff base. Beads prebound to cysteine and treated with 30% ethyl nitrite (EtONO) (22) became pink (Figure 1B); bead-bound L-CSNO was confirmed (23). Proteins were loaded, unbound proteins eluted, and L-CSNO on the beads was reduced (β-mercaptoethanol). L-CSNO–binding proteins were eluted, separated by SDS-PAGE (Figure 1C), and identified by LC-MS.

Thirty-five membrane protein classes were identified in Method 1 (Supplemental Table 1; supplemental material available online with this article; https://doi.org/10.1172/jci.insight.134174DS1) and Method 2 (Supplemental Table 2). Specific proteins identified by both methods included syntaxin binding protein 1, vacuolar ATPase G2, and voltage-gated K^+^ channel (Kv) proteins, including both Kvβ2 (KCNAB2) and Kv1.1 (KCNA1). Kv1.2 and -1.6 (KCNA2 and -6) were also identified by Method 1 (Supplemental Table 1), and Kv2.2 (KCNB2) was identified by Method 2 (Supplemental Table 2). We then showed that Kv1.1 binding to the L-CSNO affinity column was ablated by genetic KCNA1 (Kv1.1) deletion (Figure 1D).

**Figure 2:**
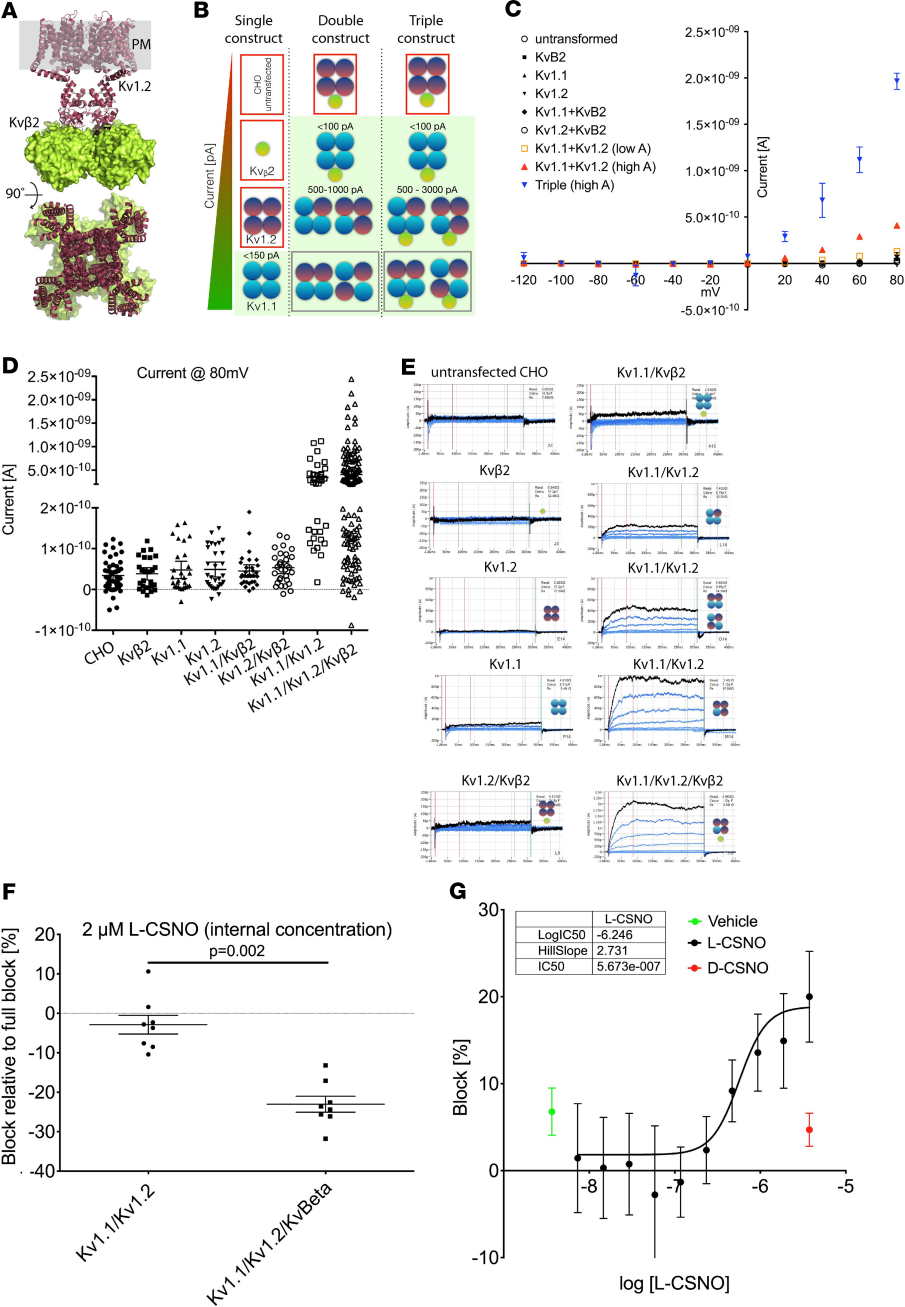
SyncroPatch analysis of the Shaker channel constructs. (**A**) Kv1.2/Kvβ2 crystal structure (PDB 2A79). The homotetramer of Kv1.2 is in the brown ribbon; Kvβ2 is the green surface; the plasma membrane (PM) is gray. (**B**) Schematic drawing of the individual cell lines after transfection with Kv1.1, Kv1.2, and Kvβ2. Not all cells will express all proteins; this schematic shows all permutations, with the corresponding experimental patch clamp results. Icons surrounded by red boxes show no conductance, while icons with a light green background show specific K^+^ channel activity. Gray boxes indicate possible conformations that cannot be distinguished based on conductance. (**C**) Conductance characteristics of the different cells lines upon voltage gate clamping from –120 mV to +80 mV (*n* = 204 single-cell patch clamp studies, with 20 untransformed, 20 Kvβ2 alone, 26 Kv1.1 alone, 28 Kv1.2 alone, 28 Kv1.1/Kvβ2, 25 Kv1.2/Kvβ2, 27 Kv1.1/Kv1.2, and 61 triple-expressing). Current at each voltage above 20 mV is greater for the triple-overexpressing than for the other cells.) (**D**) Conductance of each cell line at +80 mV resting potential. Note that only Kv1.1/Kv1.2 and Kv1.1/Kv1.2/Kvβ2 show currents >500 pA. *n* is the same as described in **C**. (**E**) Examples of individual traces, showing 11 sweeps corresponding to the ramping protocol for different cells. Black is +80 mV. All traces are on the same scale. (**F**) Effect of L-SNOC on K^+^ current block in either the double or triple cell line. *n* = 8 cells in each condition; *P* = 0.002 by Mann-Whitney rank-sum test *t* test. (**G**) IC_50_ determination of L-CSNO on triple cell line with currents >500 pA represented as % block (error bars represent a confidence level of 95% [CL 95]) compared with TEA full block. There is no block with either vehicle (green) or D-CSNO (red). *n* = 8 in the L-CSNO group and *n* = 8 in the D-CSNO group. At concentrations greater than 500 nM (*n* = 236 points analyzed), inhibition by L-CSNO is greater than inhibition by D-CSNO (*P* = 0.0001 vs. D-CSNO and *P* = 0.0048 vs. vehicle, by Kruskal-Wallis test). Data are presented as median ± 95% CI.

Kv channel complexes are typically multimers of Kvα and Kvβ proteins (24, 25) (Figure 2A). To determine whether the L-CSNO–Kv interactions observed by chromatography/LC-MS were physiologically relevant, we studied effects of L-CSNO and D-CSNO on CHO cells overexpressing Kvα (Kv1.1 and Kv1.2) and Kvβ2 proteins (Figure 2, B and C, and Supplemental Figure 1). L-CSNO, but not D-CSNO, inhibited the K^+^ currents elicited by slow voltage ramps in these overexpressing cells (Figure 2B and Supplemental Figure 1). Cells expressing all 3 proteins were most affected (Figure 2, E and F). Intracellular Kvβ2 was required for optimal inhibition by L-CSNO (Figure 2, E–G). One percent to 2% of the extracellular L-CSNO entered the cells through the L-amino acid transporter (L-AT) (19) during the 1 minute incubation (Supplemental Figure 2). Dose-response studies revealed an intracellular IC_50_ for L-CSNO of approximately 500 nM; vehicle and D-CSNO were inactive (Figure 2F).

**Figure 3:**
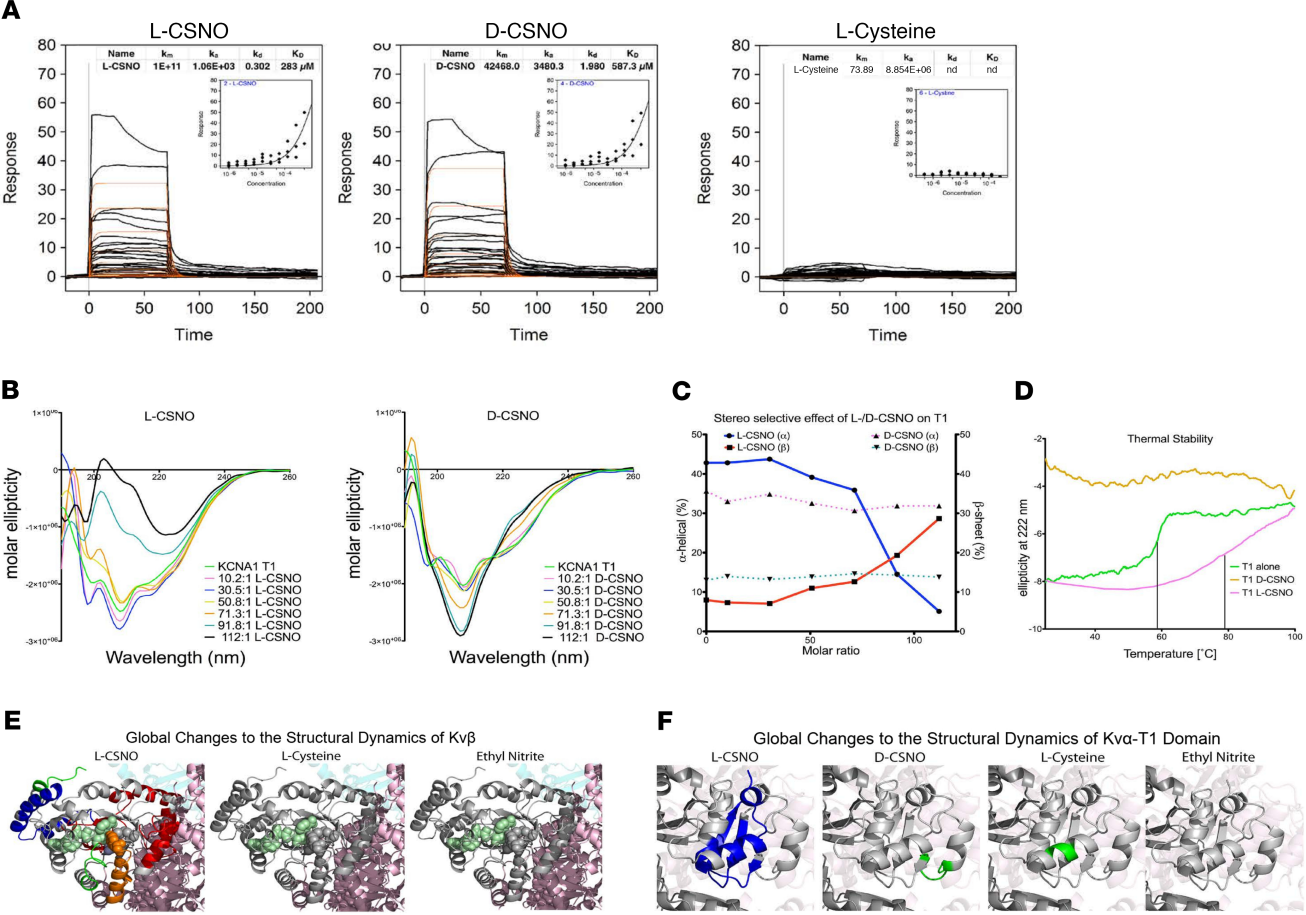
L-CSNO binding to Kv proteins. L-CSNO has unique binding interactions with the intracellular Kv proteins Kv1.1α T1 (**A–E**) and Kvβ2 (**F**) (see also Supplemental Figure 3). (**A**) The SPR binding response of L-CSNO, D-CSNO, or l-cysteine to Kv1.1α T1. The binding isotherm for each protein-ligand interaction is represented in the inset of each graph. *n* = 25 measurements each. Data are mean ± SEM.(**B**) CD analysis of Kv1.1α T1 in the presence of increasing amounts of L-CSNO and D-CSNO. *n* = 7 measurements each. (**C**) Secondary structure changes of Kv1.1α T1 upon titration with L-CSNO or D-CSNO. (**D**) Substrate stereoselectivity and thermal stability of Kv1.1α T1. Melting temperature increases by 20°C upon L-CSNO binding to Kv1.1α T1. k_m_, Michaelis constant; k_a_, association constant; k_d_, dissociation constant; KD, affinity. (**E**) Differential deuterium uptake after a 15 minute pulse between the unliganded Kvβ and in solution with L-CSNO, D-CSNO, l-cysteine, or EtONO. Blue peptides represent a greater than 20% rigidification; green peptides represent between 0% and 20% rigidification. (**F**) Differential deuterium uptake after a 1 minute pulse between the unliganded Kv1.1α T1 and in solution with L-CSNO, D-CSNO, or l-cysteine. Blue peptides represent a greater than 20% rigidification. Green peptides represent between 0% and 20% rigidification, and gray peptides represent no significant rigidification. NADPH is shown in teal spheres.

We next studied directly the interactions of L-CSNO with isolated monomers of Kvβ2 and the intracellular subunit (T1) of Kv1.1. By surface plasmon resonance spectroscopy (SPR), circular dichroism (CD), and hydrogen-deuterium exchange (HDX), L-CSNO — but not D-CSNO or l-cysteine — modified the structure of the Kv1.1 subunit, T1, that interacts with Kvβ proteins (Figure 3). Moreover, L-CSNO, but not L-cysteine or EtONO, also modified isolated Kvβ2 as assessed by SPR and by HDX (Figure 3 and Supplemental Figure 3). Surprisingly, L-CSNO did not *S*-nitrosylate Kvβ2 as assessed by direct chemiluminescence assay of the purified protein (Supplemental Figure 4); nor did it *S*-nitrosylate any of the 3 Kv proteins in the Kv1.1/Kv1.2/Kvβ2-overexpressing CHO cells, as assayed by biotin substitution followed by LC-MS (26).

**Figure 4:**
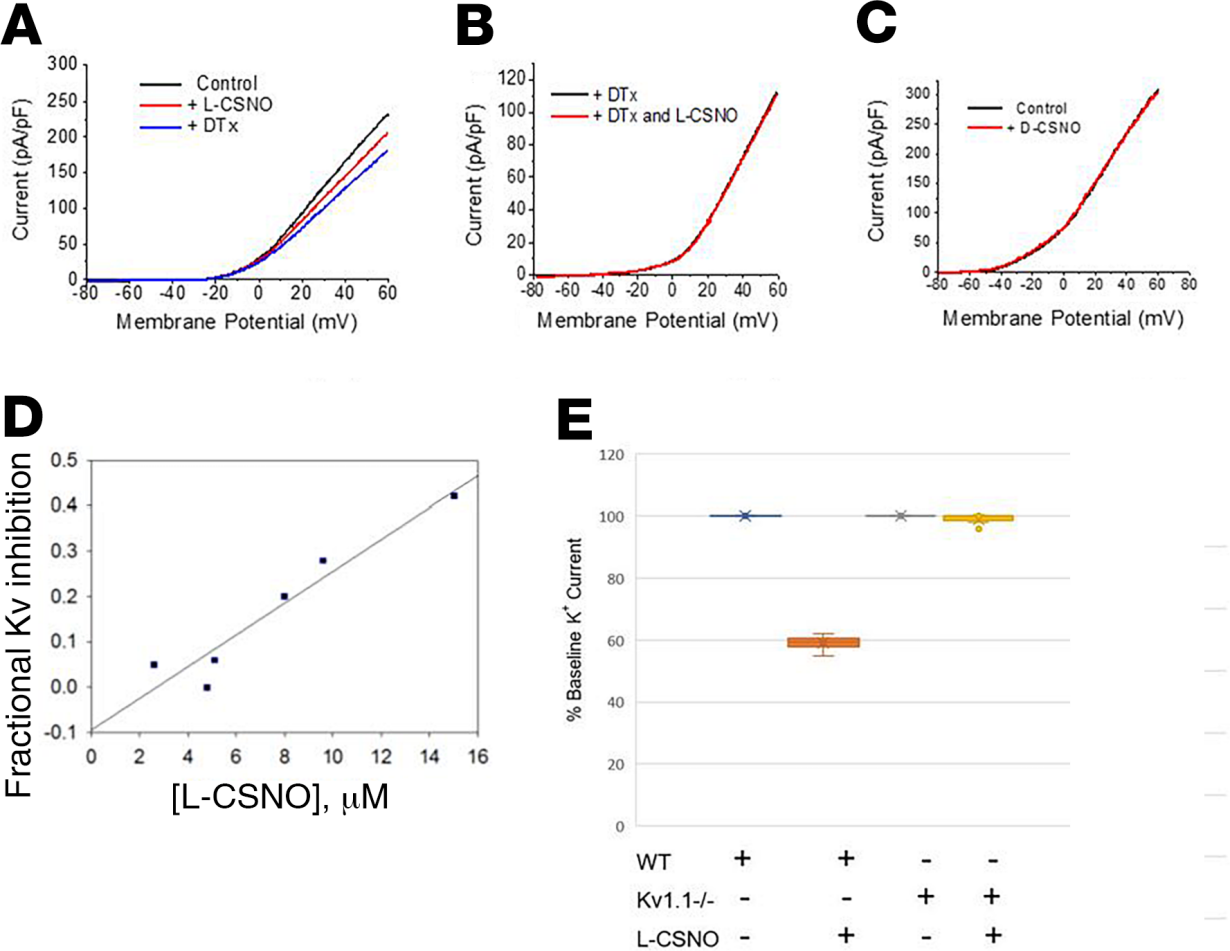
Voltage-sensitive K^+^ currents are inhibited by L-CSNO and hypoxia. (**A**). Extracellular L-CSNO reduced K^+^ current elicited by a slow voltage ramp from –80 to +60 mV in newborn rat petrosal ganglion cells studied by patch clamp. (**B**) After DTx, L-CSNO produced no further reduction in K^+^ current (*n* = 3 animals each). DTx blocks approximately 20% of the total K^+^ current (28). (**C**) D-CSNO had no effect on K^+^ current (*n* = 4, 100 μM to 1 mM). (**D** and **E**) As in rat neurons, L-CSNO inhibited DTx-sensitive voltage-gated K^+^ current relative to inhibition by subsequent DTx in WT murine ganglia in a dose-dependent fashion (**D**; *n* = 6; Inh_max_ = 0.4), but (**E**) maximal doses (>20 μM) had no effect in Kv1.1^–/–^ murine neurons (*n* = 4 Kv WT neurons and *n* = 6 Kv1.1^–/–^ neurons; *P* < 0.05 by ANOVA).

It has been established that Kv channels are relevant to respiratory control (27–29). To determine whether these stereoselective L-CSNO–Kv interactions were relevant in mammalian physiology, we began by studying ex vivo whole-cell petrosal ganglion preparations of respiratory control neurons from rats (Figure 4, A–C) and WT mice (Figure 4, D and E). L-CSNO, but not D-CSNO, inhibited Kv currents in a dose-dependent fashion (Figure 4D). Whereas Kvβ2^–/–^ mice have a complex phenotype (30), respiratory responses of Kv1.1^–/–^ mice can be studied by plethysmography. These mice have a decreased ventilatory response to hypoxia and to normoxic recovery (27). We therefore studied the petrosal ganglion Kv responses of Kv1.1^–/–^ mice, which have measurable but attenuated Kv currents. Unlike in WT mice, currents in Kv1.1^–/–^ mice could not be inhibited by L-CSNO (Figure 4E).

**Figure 5:**
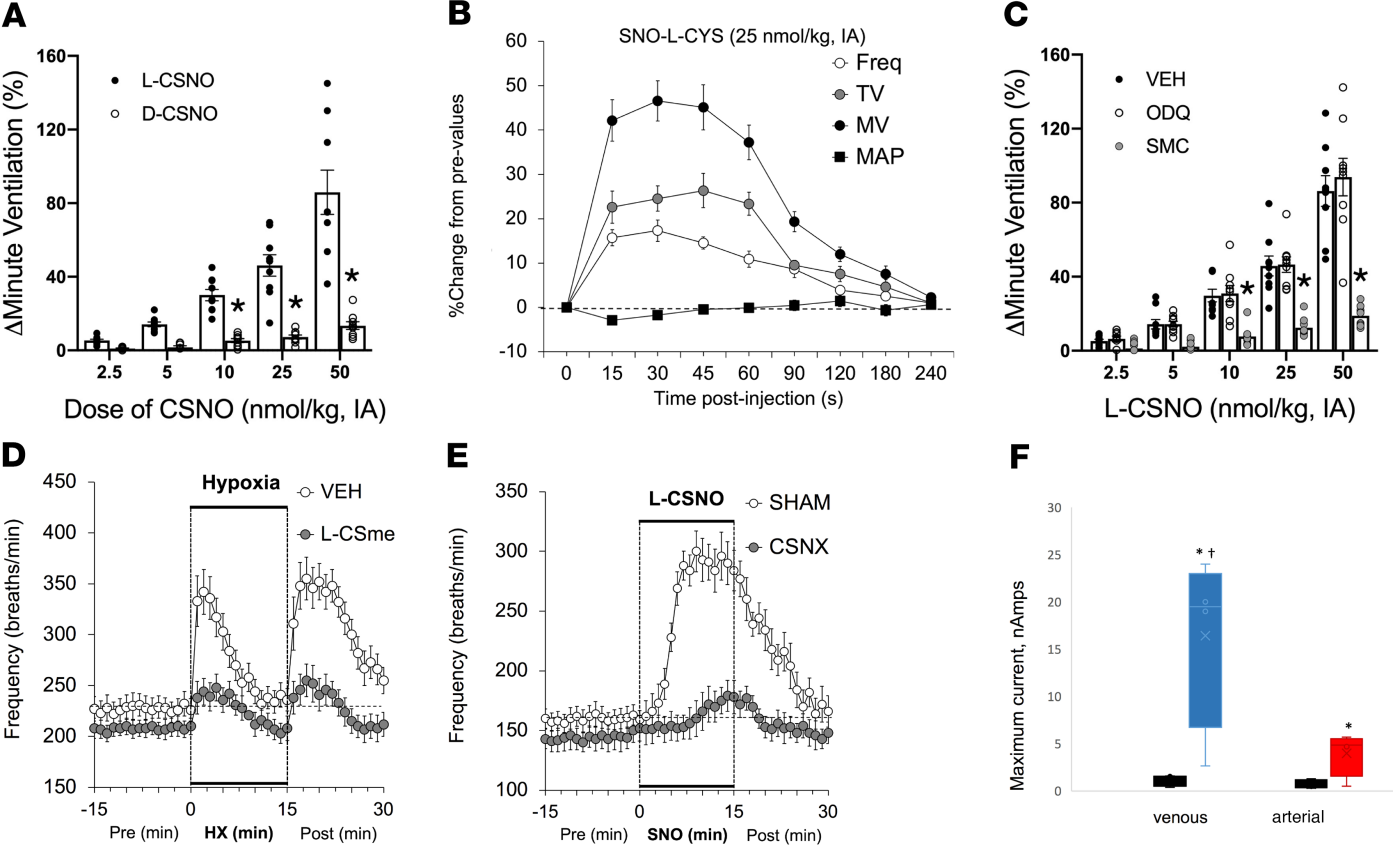
Stereoselective biological activity of the endogenous ligand, L-CSNO. (**A**) Maximal increases in V_E_ elicited by arterial injections of L-CSNO and D-CSNO in conscious Sprague-Dawley rats (*n* = 9). *P < 0.05, by ANOVA on ranks; †*P* < 0.05, D-CSNO versus L-CSNO. Mean ± SEM. IA, intra-arterial. (**B**) Changes in frequency of breathing (Freq), TV, V_E_ [minute ventilation, MV]), and MAP elicited by a 25 nmol/kg dose of L-CSNO (*n* = 9 each; mean ± SEM at each time point). (**C**) Maximal increases in V_E_ elicited by L-CSNO in Sprague-Dawley rats receiving infusions of vehicle (VEH; 0.1% DMSO in saline, 20 μL/min), ODQ (2 mg/kg bolus followed by 50 μg/kg/min, i.v.), or S-methyl-L-cysteine (SMC or L-CSMe) (10 μmol/kg/min, i.v.). *n* = 8–10 animals/experiment. *P < 0.05, SMC versus VEH or ODQ. Mean ± SEM. (**D**) Mice preinstrumented with arterial catheters at the level of the CB were treated with the L-CSNO congener L-CSMe (filled circles) or vehicle (open circles), then exposed sequentially to 10% oxygen or room air. L-SMC almost completely ablated the normal hypoxic response and recovery (*n* = 9 each; *P* < 0.001). At each time point, mean ± SEM. (**E**) The effect of L-CSNO is mediated by the CB. Mice underwent CSN (CSNO transection and carotid artery cannulation (as in Figure 5D). After a 3 week recovery, they were exposed to infusions of L-CSNO. Mice with intact CSN (SHAM, open circles) had a normal response to carotid L-CSNO infusion, while those with CSN transection (closed circles) did not (*n* = 12 each; mean ± SEM; *P* < 0.001 by ANOVA on ranks). (**F**) Detection of L-CSNO in blood samples. *n* = 3 each; median and CI. *P < 0.05, significant value; †*P* < 0.05, venous versus arterial, by ANOVA on ranks.

To study whole-animal responses, we performed plethysmographic studies of awake, preinstrumented rats receiving intra-arterial carotid body (CB) CSNO injections. L-CSNO, but not D-CSNO, increased minute ventilation (V_E_) (both respiratory rate [RR] and tidal volume [TV]; Figure 5A and Supplemental Figure 5). This effect of L-CSNO was not related to any change in hemodynamics (Figure 5B) and was inhibited by the L-CSNO congener L-CSMe (20) (Figure 5C and Supplemental Figure 6). The actions of L-CSNO were NO *independent*: (a) NO depressed respiration, consistent with previous data (31); and (b) NO, but not L-CSNO, activity was blocked by sGC inhibition (Figure 5C and Supplemental Figures 6 and 7). To confirm that L-CSNO signals increased V_E_ at the level of the CB, we perfused the CB in vivo with the L-CSNO congener that blocks L-CSNO bioactivity, L-CSMe. Remarkably, L-CSMe almost completely inhibited the ventilatory response to hypoxia (Figure 5D). Moreover, we infused L-CSNO at the level of the CB in rats that had previously undergone carotid sinus nerve transection (CSN). There almost no response to CB L-CSNO in the CSN-transected animals (Figure 5E).

Next, we asked whether L-CSNO is present endogenously. Several previous studies have demonstrated the presence of GSNO and NO-modified proteins, but the GSNO catabolic product L-CSNO is somewhat elusive because of its lability (17, 32). We have recently developed a sensor (33) that can be adapted to be specific for L-CSNO through coupling with an anti–L-CSNO antibody (34, 35). Negative controls for this sensor included *S*-nitrosocysteamine, GSNO, l-cysteine, and *S*-nitrosoalbumin (Supplemental Figure 8). 100-fold excess of the latter two L-CSNO–containing compounds gave an attenuated signal, but these mid-micromolar levels are not present endogenously (2, 5, 7). Assays performed immediately after phlebotomy revealed that L-CSNO, barely detectable in normoxic arterial blood, was present at a high level in deoxygenated blood (Figure 5F), consistent with known *S*-nitrosothiol biochemistry (1, 6–8). Thus, this small molecule present in hypoxic blood stereoselectively signaled increased V_E_ at the level of the CB.

## Discussion

Using an unbiased proteomic approach and several validation steps, we have identified a class of cell membrane–associated proteins that are structurally and functionally modified by L-CSNO. Pharmacological evidence had suggested the existence of such a target for decades (1, 12–18, 31). Specifically, L-CSNO has a number of bioactivities in mammalian central and peripheral neurons, in vascular beds, in muscle, and in blood; but D-CSNO fails to recapitulate these bioactivities (1, 12–18, 31). These effects are, for the most part, NO and sGC independent, and they can be inhibited by the L-CSNO congeners L-CSMe and L-CSφ. Here, we have studied the interaction of L-CSNO with Kv proteins, a class of proteins identified using both L-CSNO affinity methods. In particular, we have focused on the interaction of L-CSNO with Kvβ2 and the Kv1.1 because (i) these specific Kv proteins were identified in our proteomic screens; (ii) they represent both the transmembrane (Kv1.1) and intracellular regulatory (Kvβ) domains of Kv complexes (24, 25); (iii) they are common components of several of the Kv heteromultimeric voltage-gated potassium channels in physiology; and (iv) one of their roles in physiology is control of respiration, an area of interest of our laboratories (1, 27, 36, 37). Additional proteins from the proteomic analysis may ultimately prove to be of interest as well.

L-CSNO interacted specifically with Kv proteins. This was evident from both the L-CSNO gel chromatography and the L-CSNO column affinity chromatography (Figure 1). In follow-up experiments, L-CSNO affinity column chromatography confirmed that L-CSNO bound Kv 1.1 from WT mice but not from Kv 1.1^–/–^ mice. Subsequent patch clamp analyses confirmed that L-CSNO inhibited voltage-gated potassium current in cells expressing the Kv 1.1/Kv1.2/Kvβ2 complex (Figure 2). Interactions with Kv proteins were inhibited by L-CSNO congeners L-CSMe (Figures 1 and 5) and L-CSφ (Figure 1). They were minimally replicated by D-CSNO in CSNO-Kv binding experiments (Figure 3) or in patch clamp experiments (Figure 2). Effects on L-CSNO binding to Kv proteins were not replicated by the NO donor and nitrosylating agent EtONO (Figure 3); indeed, 2 separate assays showed that L-CSNO did not chemically modify Kv proteins (Figure 4). Taken together, these structural and functional data show that interaction with Kv could be a mechanism by which L-CSNO stereoselectively affects biology.

However, it remained to be determined whether L-CSNO–Kv interactions could be relevant to physiology at the whole-organ level. We performed studies on respiratory control because Kv 1.1^–/–^ mice have disordered respiratory control (27), and additional Kv channels are relevant to a range of disorders of control of breathing (28, 29). Hypoxia decreases voltage-gated potassium currents in the CB (29). Note that our CSN transection studies here have shown that the CB is important for sensing hypoxia. L-CSNO stereoselectively signals increased V_E_ in the nucleus tractus solitarius (1), while NO itself depresses respiration (31). We thus asked whether L-CSNO would function as a stereoselective hypoxia mimetic at the level of the CB. Therefore, we studied voltage-gated K^+^ currents in preparations of respiratory control neuron complex isolated from the rat, including petrosal and nodose ganglia, together with innervation from the CB (27). Patch clamp techniques confirmed that α-dendrotoxin–inhibitable (DTx-inhibitable) Kv currents were present in these preparations, as we have previously reported. L-CSNO, but not D-CSNO, partially recapitulated the effect of DTx (Figure 3). We then addressed the challenge of performing dose-response studies using the labile molecule L-CSNO in this assay system: because of the flow rate, substrate can be lost in the tubing before reaching the neuron. We therefore withdrew the perfusion medium and froze it in liquid nitrogen to measure the actual L-CSNO concentration at the time of inhibition. Then, to confirm genetically that Kv channels are inhibited by L-CSNO, we also studied murine petrosyl ganglia preparations. Kv currents were inhibited by L-CSNO in WT mice but not in Kv^–/–^ mice (Figure 3).

Next, we directly measured the effect of L-CSNO on CB respiratory stimulation in vivo. To do this, we preinstrumented rats with a cannula tip just distal to the level of the CB and studied awake, freely moving animals in a plethysmograph. Use of rats is optimal for these studies, because their size allows to for precise catheter placement (2). L-CSNO, but not D-CSNO, administered at the level of the CB increased V_E_ by increasing both TV and RR (Figure 5 and Supplemental Figure 6), an effect ablated by CSN transection (Figure 5E). The effect was not secondary to a change in blood pressure (Figure 5B). Like other stereoselective L-CSNO effects, the ventilatory effect was neither NO nor sGC dependent, and it was inhibited by L-CSMe, which inhibits Kv–L-CSNO interactions (Figure 5). Of note, L-CSMe administered at the level of the CB also inhibited hypoxia’s effect of increasing V_E_ (Figure 5D), confirming the relevance to the normal hypoxic response.

Our data are consistent with previous reports that GSNO and related signaling molecules are formed in deoxygenated blood (1, 6–8). However, L-CSNO is labile (17, 32), and this is the first study to our knowledge specifically of L-CSNO in deoxygenated blood, using a sensitive and specific capacitance-based immunosensor. Note that L-CSNO and GSNO can also be produced by other metalloproteins, such as NOSs (3–5, 9), and that NOS isoforms are expressed in the CB (38). Indeed, L-CSNO produced by NOS has been proposed to be concentrated and stabilized in synaptic vesicles like other neurotransmitters (our unpublished observations). Because L-CSNO interacts with intracellular components of the Kv complex, L-CSNO formed either in blood or presynaptically would need to cross the target cell plasma membrane. As previously reported for other cells, time-dependent L-CSNO uptake across the plasma membrane in CHO cells involves the LAT (ref. 19; Supplemental Figure 2), and an element of the stereoselectivity of L-CSNO activity could involve regulation of its uptake. However, the bioactivities described here for L-CSNO are NO independent (Figure 5); and the SPR, HDX, biochemical, and inhibitor data suggest that the interaction of the L-CSNO moiety with target Kv proteins is also isomer specific.

**Figure 6:**
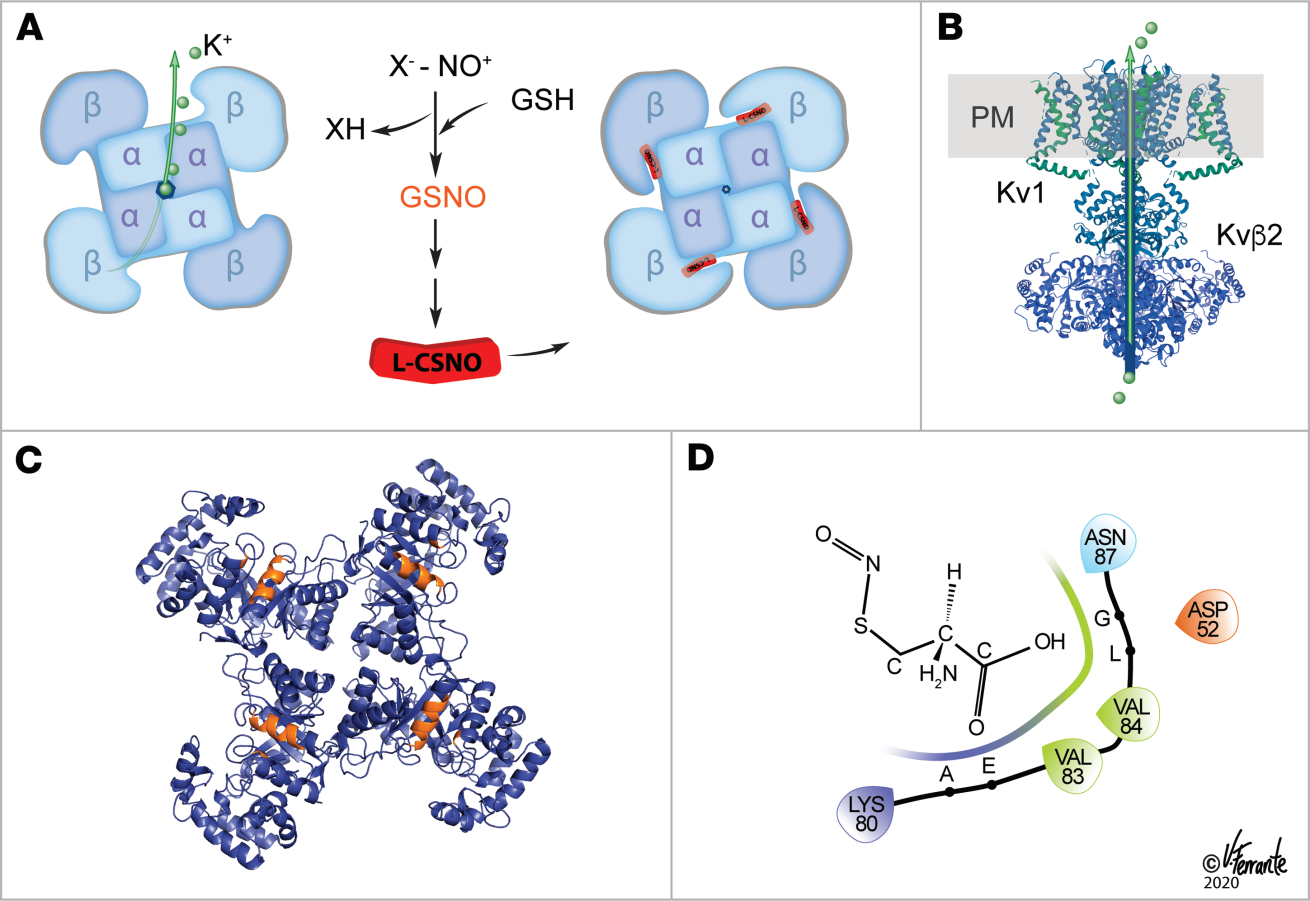
L-CSNO inhibition of voltage-gated K^+^ current. (**A**) Top view: Schematic showing L-CSNO’s effect of altering the structure of the Kv multimer. The HDX data (Figure 3 and Supplemental Figure 3) show that the tertiary structure of Kvβ2 is altered by L-CSNO binding to make the protein more concave, particularly at the base near the site of its interaction with Kvα proteins. Kv1.1 T1 is also affected (Figure 3 and Supplemental Figure 3), but patch clamp data suggest that the interaction with Kvβ2 is essential for a functional effect (Figure 2). L-CSNO is formed from GSNO by γ-glutamyl transpeptidase and downstream dipeptidases (1), signaling increased V_E_ (1). GSNO is formed, in turn, by NOS activation, oxyhemoglobin desaturation, ceruloplasmin and other metalloproteins capable of transferring NO^+^ equivalents to thiolate anions. Uniquely, NO is not transferred to the Kv proteins. D-CSNO does not share the activity of L-CSNO (Figures 2–5), though both isomers form NO at the same rate (20). (**B**) Lateral view of the Kvα/Kvβ complex in the plasma membrane, as in Figure 2A: side view of K^+^ exit from the cell. (**C**) Based on HDX, approximate sites of L-SNO binding in the 4 Kvβ2 subunits. (**D**) In silico estimates of L-CSNO interaction with Kvβ2 amino acids using ChemDraw and Schrödinger Maestro software. Reproduced with permission from V. Ferrante.

It bears mentioning that L-CSNO signaling is reminiscent of other signaling pathways. First, L-CSNO itself is quite labile (17, 32), like lipid mediators such as prostacyclin, which has a half-life of 42 seconds in vivo (39), and acetylcholine (AcChol), which has a half-life of microseconds in the neuromuscular junction. L-CSNO has a half-life of approximately 84 seconds, in the presence of membranes (40), but its inorganic aqueous and lipid stability is likely less important to its bioactivity than its transmembrane transport through LAT (19). As with prostacyclin and AcChol, L-CSNO lability is likely of central importance to its lack of toxicity: if it were not labile, sustained intravascular or neuronal activity could result in life-threatening hypotension and other undesirable consequences. Second, it is produced in mammals by breakdown of GSNO (1, 2, 11) (Figure 6A). There is a regulated pathway by which GSNO is bioactivated (1–5, 11), and L-CSNO is one important effector end product. This pathway shares features with the cysteinyl leukotriene pathway. The effect of blocking GSNO conversion to L-CSNO on ventilatory drive is known. For example, genetic or pharmacologic ablation of γ-glutamyl transpeptidase, the enzyme mediating the first step in conversion of GSNO to L-CSNO, prevents the ventilatory effect of GSNO (1). Here we have identified the molecular target of the downstream effector molecule, L-CSNO. Thus, a receptor-based system for a labile product of thiol-modified glutathione cleavage is not without analogies in biology. Using data from HDX, coupled with ChemDraw-generated (PerkinElmer) L-CSNO and Kvβ2 structures from Protein Data Bank and Schrödinger Maestro, we modeled potential binding of L-CSNO to Kvβ2 (Figure 6, B–D). We anticipate that the techniques we have developed here for affinity binding may be useful for identifying additional target proteins.

To the best of our knowledge, the ability of L-CSNO to change Kv structure and function without NO transfer is unique in nitrogen oxide signaling (2). Various redox forms of oxidized nitrogen can transfer NO equivalents to metals as well as to sulfur-, oxygen- and nitrogen-containing moieties to cause specific bioactivities or toxicities (2, 41–44). In the case of L-CSNO–Kv interaction, however, our evidence to date demonstrates that the target protein is structurally modified without NO transfer. It is also of interest that there is overlap between nitrogen oxide–related pathology and Kv-related pathology, and our findings provide a potential explanation for this convergence. In this regard, these findings might be exploited to study treatments for Kv-associated conditions (27, 30), because both endogenous agonists and pharmacological antagonists of the Kv–L-CSNO interaction are described here.

## Methods

### Materials.

Reagents were purchased from MilliporeSigma unless otherwise noted. *S*-nitrosothiols were prepared from reduced thiols by reaction with acidified nitrite, followed by alkalinization to pH 7.4 using NaOH in deionized water as previously described (23, 45). Purity was analyzed by Saville reaction (36) after synthesis and before use. Molecules were stored at –80°C until use.

All rats were from Harlan. All mice were from the Jackson Laboratory.

### Isolation of brain membrane fractions for affinity chromatography.

Excised C57BL/6 mouse cortex was homogenized in HEPES buffer (250 mM HEPES-NaOH, pH 7.7, 1 mM EDTA, and protease inhibitor cocktail [MilliporeSigma]) with a Dounce homogenizer and maintained on ice (17). Homogenates were separated into cytosolic and membrane fractions with a fixed-angle ultracentrifuge (rotor is 70.1Ti; Beckman Coulter, Optima L-90K Ultracentrifuge) at 105,000*g* for 40 minutes (4°C). Membrane fractions were removed by pipetting and maintained in HEPES buffer until used for identification of L-CSNO binding partners.

### Protein isolation: Method 1, L-CSNO binding to proteins after native PAGE separation.

The mouse brain membrane fraction (described above) was resuspended in HEPES buffer. A BCA (Pierce) protein assay was performed, and 50 μg protein was run on 2 native PAGE gels (TGX, Bio-Rad). One gel was incubated for 30 minutes in the dark with 50 μM L-CSNO in *S*-nitrosothiol buffer (10 mM Tris-HCl, pH 6.0, 150 mM NaCl). The second gel was incubated for 30 minutes in the dark with 50 μM L-CSNO and 100 μM each L-CSφ and L-CSMe in *S*-nitrosothiol buffer. Each gel was then rinsed with water and incubated in the dark with 40 μM DAF2 (Cayman Chemicals) for 10 minutes in the dark (room temperature) (21). Gels were then imaged on Chemidoc (Bio-Rad) using the fluorescein setting, and bands were cut out for mass spectrometry proteomics (see below) that were seen without, but not with, the *S*-methyl– and *S*-phenyl–substituted cysteine coincubations (20).

### Protein isolation: Method 2, L-CSNO affinity chromatography.

L-CSNO affinity columns were prepared as follows. l-Cysteine was coupled to AminoLink Plus (Pierce/Thermo Fisher Scientific) resin according to the manufacturer’s protocol. In control experiments, AminoLink Plus was used without reaction with l-cysteine. Briefly, l-cysteine was dissolved in coupling buffer A (0.1 M sodium citrate, 0.05 M sodium carbonate, pH 10) at a concentration of 100 mM and incubated with 0.4 mL previously washed AminoLink resin in a small (0.5 mL) column for 4 hours at room temperature. The resin was then washed with coupling buffer B (0.1 M phosphate. 0.15 M sodium chloride, pH 7.2) and incubated for 4 hours at room temperature with 100 mM cyanoborohydride in coupling buffer B. Active sites were then blocked with quenching buffer (1 M Tris-HCl, pH 7.4). Resin was then washed with 1 M NaCl and then incubated for 10 minutes with 30% EtONO in ethanol at room temperature in the dark (22). EtONO modifies the thiol of the L-cysteine bound to the column at the amine. As a result, the column turns pink (Figure 1B; ref. 23). It was then washed with 100% ethanol, followed by 1 M NaCl. Excised mouse brain homogenate was briefly centrifuged, and the supernatants were then incubated with prepared AminoLink columns for 30 minutes at room temperature. The columns were then washed with wash buffer, and bound protein was eluted with Laemmli buffer containing β-mercaptoethanol and elution buffer (0.1 M glycine, pH 3.5). Eluate was concentrated and used for SDS-PAGE gel (TGX, Bio-Rad), followed by Coomassie staining. Bands found in samples from +L-cysteine columns but not in those from –L-cysteine columns were excised and analyzed further by MS proteomics (see below).

### LC-MS proteomics.

Proteolysis: Protein was digested from in-gel (Method 1) or in-the-column eluate (Method 2) using 100 ng of modified trypsin (Promega) overnight at 37°C. LC-MS analysis: LC-MS experiments were carried out on the Orbitrap Elite mass spectrometer (Thermo Electron) interfaced with a Waters nanoACQUITY UPLC system. Approximately 400 ng proteolytic peptide mixture was loaded onto a trap column (180 μm × 20 mm packed with C18 Symmetry, 5 μm, 100 Å; Waters) to pre-concentrate sample and wash away excess salts. Reverse-phase separation was performed on a reversed-phase column (75 μm x 250 mm nano-column, packed with C18 BEH130, 1.7 μm, 130 Å; Waters) using a gradient of 2%–47% mobile phase B (100% acetonitrile (ACN)/0.1% formic acid) and mobile phase A (100% water/0.1% formic acid) over 64 minutes at 37°C and flow rate of 300 nL/min. Peptides eluting from the column were directed to a nano-electrospray source with capillary voltage of 2.5 kV. All mass spectra were obtained from data-dependent experiments. MS and MS/MS spectra were acquired in the positive ion mode, with the full scan MS recorded for eluted peptides (*m/z* range of 350–1700) in the Fourier transformed (FT) mass analyzer at a resolution (*R*) of 120,000, followed by MS/MS of the 20 most intense peptide ions scans in the ion trap mass analyzer. Data analysis: The resulting MS/MS data were searched against mouse protein database using the Mascot search engine (Matrix Science). In particular, MS/MS spectra were searched for tryptic peptides using mass accuracy values of 8 ppm and 0.7 Da for MS and MS/MS scans, respectively, with the allowed variable modifications including carbamidomethylation for cysteine and oxidative modifications for methionine amino acids.

### Analysis of the candidates for biological relevance from the proteomics data.

Mascot analysis (as above) of bands from Method 1 revealed more candidate proteins than Method 2. Beginning with Method 1 proteins, data were systematically analyzed by members of our research team to identify proteins for which there was (a) low likelihood of representing a contaminant (keratins, for example, were excluded); (b) concordance between replicate experiments; (c) likely membrane association; and (d) potential for membrane-associated signalling function. These were organized according to protein class (Supplemental Table 2) with concordance between methods highlighted (Supplemental Tables 2 and 3) and reviewed by the research team as a whole to ensure that all possible candidates were included.

The proteomics data have been deposited in Mendeley Data (https://data.mendeley.com/datasets/v6gpnpxfm8/1).

### Cell lines and reagents for the Kv1.1/Kv1.2/Kvβ2 overexpression system.

CHO K1 (CCL-61) cells were purchased from ATCC and cultured in F-12K medium (Gibco) supplemented with 10% FBS (Hyclone). Lenti-X 293T cells (Takara Bio) were grown in DMEM high glucose (Hyclone) in 10% FBS. One day before virus packaging (see below), Lenti-X 293T cells were switched to medium containing tetracycline-free FBS (Takara). Geneticin (Gibco), puromycin (Gibco), and hygromycin (Invitrogen) were used for selection.

### Lentivirus packaging and transduction.

Lentiviral (LV) vectors (pLV-Puro-CMV-hKCNA2 [NM_004974.3], pLV-Neo-CMV-hKCNA1 [ORF009793], and pLV-Hygro-CMV-hKCNAB2 [ORF024513]) constructed by VectorBuilder were used for stable expression of KCNA2, KCNA1, and KCNAB2, respectively, in CHO K1 cells. Lentivirus was produced in Lenti-X 293T cells following the Lenti-X Packaging Single Shots VSV-G (Clontech Laboratories) protocol. Cells were infected with LV following the Lenti-X Lentiviral Expression Systems protocol. To enhance transduction, infectious medium was supplemented with polybrene (MilliporeSigma). To create double and triple Kv cell lines, CHO K1 cells were transduced in series.

### High-throughput patch clamp analyses.

Cell suspensions were prepared shortly before being loaded into a Synchropatch384PE (Nanion Technologies). Cells were kept at 10°C under constant shaking. All compound solutions were freshly prepared before the run of each plate, and if needed the pH was adjusted to pH 7.0. Compounds were diluted in *N*-methyl-d-glucamine 60 (NMDG-60) buffer (in mM; 80 NaCl, 60 NMDG, 4 KCl, 5 d-glucose, 2 CaCl_2_, 1 MgCl_2_, 10 HEPES; pH 7.4). Full Kv block solution was composed of 20 mM tetraethylammonium (TEA) and 10 mM 4-aminopyridine (4-AP) in NMDG-60. Voltage protocol: All whole-cell recordings were performed on the SyncroPatch 384PE. Data acquisition and analysis were carried out with PatchControl 384 and DataControl 384, respectively (Nanion Technologies). All recordings were collected using planar borosilicate glass patch clamp chips in a 384 microtiter plate format with a single hole per well and classified as high-resistance plates. For recordings, we used standard intracellular solution (containing in mM): 10 KCl, 110 KF, 10 NaCl, 10 EGTA, 10 HEPES; pH 7.2; and NMDG-60 extracellular solution (consisting of, in mM): 80 NaCl, 4 KCl, 2 CaCl_2_, 1 MgCl_2_, 5 glucose, 60 NMDG, 10 HEPES; pH 7.4. Prior to the electrophysiological measurements, cells were harvested by trypsinization. The cell suspensions (200,000–320,000 cells/mL) were kept in the dedicated cell reservoir at 10°C and shaken at 200 rpm in standard external solution. Each cell preparation was used for no longer than 2 hours. For experiments, 10 μL cell suspension (2,000–3,200 cells) was added to each well. Only cells with a seal resistance R_seal_ >500 MΩ and currents >100 pA were considered for subsequent analysis. Current was elicited using 300 ms voltage steps, V_hold_ = −100 mV to +40 mV at 0.2 Hz. To further distinguish between the triple-construct and double-construct currents, an additional filter was applied where only cells with currents ≥500 pA were selected for further analysis. By applying this additional filter a clear difference upon compound addition was observed between the triple construct and double construct currents.

### Cellular L-CSNO uptake experiments.

CHO cells overexpressing Kv1.1, Kv1.2, and Kvβ2 as above were grown on coverslips to 90% confluence. Twenty-four hours before experiments, cell medium was replaced with serum-free medium. CHO cells were treated (*n* = 3 each) with 100 μM CSNO, 500 μM CSNO, 1000 μM CSNO, and 1000 μM CSNO with 10 mM leucine for 1 minute (19). At time 0, 100 μL media from each sample was collected and frozen in dry ice in the dark. At 1 minute, cells were rapidly washed 3 times in PBS, pipetted dry, scraped, and frozen in the dark in dry ice. Baseline supernatant and 1 minute cell lysate were assayed using a copper-cysteine chemiluminescence assay, as described previously (46). Inside/outside CSNO ratio was calculated for each condition.

### Surface plasmon resonance studies of L-CSNO binding to Kvα1.1 T1 and Kvβ2.

Isolated, purified Kv proteins were commercially obtained from MyBiosource (Kvβ2, MBS956299; Kv1.1α T1a, MBS1265383). They were provided in 50 mM Tris pH7.5, 50% glycerol. Sample purity was verified by SDS-PAGE (Supplemental Figure 1) to be compatible with amine coupling for attachment to the SPR chip (CM5, Biacore, GE). The protein was first dialyzed overnight into 1× PBS. The protein was then passed over an EDC/NHS-activated chip at a concentration of 0.1 mg/mL for 15 minutes, depositing 7500 RU to the chip surface. 0.5 M ethanolamine in PBS was used to inactivate the remaining sites on the chip. All SPR runs were carried out at 25°C using 10 mM Tris pH 7.4, 3 mM EDTA, 0.01% P20, and 1 mg/mL soluble CM5 in triplicate. Kinetics studies were carried out at a maximum flow rate of 100 μL/min with 90 second contact time of each analyte, followed by 120 seconds dissociation. Steady-state kinetic measurements were carried out at 20 μL/min with 120 seconds contact time for each analyte, followed by 120 seconds dissociation. Sensorgrams were analyzed with Scrubber 2.0 (BioLogic Software) using the double-referencing method, subtracting a reference cell as well as buffer blanks.

### CD binding and thermal stability studies of Kv1.1α T1 with ligands.

CD studies were done using a Jasco 815 CD spectrometer at 25°C with a cuvette of 1 mm path length and a total volume of 300 μL. Titration studies with either L-CSNO or D-CSNO were carried out with different molar ratios by repeatedly titrating ligand into the cuvette. For each sample, a spectrum ranging from 190 nm to 260 nm was collected with 1 nm intervals in 3 passes, the average of the 3 passes being used. Protein sample concentration at the beginning of the measurement was 5 μM and after the final addition of ligand 3.97 μM. The molar excess of ligand to protein ranged from a 10:1 to 112:1 ratio. A buffer baseline was subtracted from each of the samples. The molar ellipticity was calculated based on a molecular weight of 18.5 kDa and the corresponding protein concentration for each sample. For thermal stability in the presence and absence of L-CSNO or D-CSNO, measurements were carried out at 222 nm to observe α-helix unfolding upon heat treatment of the sample. The temperature was raised at 0.1°C intervals starting at 25°C to 100°C, with ramp temperature of 1°C per minute.

### HDX and modeling.

Tetrameric (22.7 μM) Kvα1.1 T1 domain or tetrameric Kvβ2 (4.7 μM) in 20 mM Tris (pH 8.0) with 50% glycerol was diluted 10-fold into D2O 10 mM Tris (pD 7.4) and allowed to incubate at 25°C for 1 minute for Kv α1.1 T1 and 15 minutes for Kvβ2. This allowed amide hydrogens not protected by secondary structure, tertiary structure, or small molecule occlusion to exchange with deuterium in solution. The reaction was then quenched by diluting 5-fold into 100 mM NaH_2_PO_4_ (pH 2.4) and placed on ice to drop the temperature to 4°C. The quench solution was digested by a mixture of porcine pepsin and aspergillopepsin for 5 minutes. The peptic fragments were then loaded onto a NanoACQUITY HDX (Waters) UPLC with a C18 column and eluted with a water ACN gradient from 5% to 95% ACN over 10 minutes into a Synapt G2 (Waters) mass spectrometer to determine their mass. Peptic fragments were identified using the ProteinLynx Global Server 2.5.1 (PLGS) from Waters. We then employed 0.3 fragments per amino acid filter for all sequenced peptic fragments when analyzing sequence data in DynamX (Waters). As the amide hydrogen atoms on of the peptic fragments were replaced by deuterium atoms, the mass of the peptic fragments shifted by 1 Da per amide hydrogen (^20^NH_3_–^23^NH_3_). These data were collected in triplicate. The number of deuterium exchanged was calculated for each peptic fragment using the following equation: *D* = (*m* – *m*_0%_)/(*m* – *m*_100%_) × *N*), where *D* is the number of deuterium exchanged; *m* is the mass of the peptic fragment after incubation in deuterium; *m*_0%_ is undeuterated mass; and *m*_100%_ is the mass of a fully deuterated control peptic fragment. The fully deuterated control was made by incubating the protein in 6 M GuDCl (pD 7.4) for 2 hours before quenching the sample and analyzing the data normally.

We analyzed deuterium exchange data for Kvα1.1 T1 and Kvβ2 either unliganded or bound to 47 μM L-CSNO, 128 mM L-cysteine, or 1 M EtONO to ensure 100% binding to or nitrosylation of these proteins. The number of deuterated amide hydrogens was compared for each peptic fragment, and only fragments that showed a mean difference greater than 3 standard deviations over the majority of overlapping sequence of peptic fragment were considered to represent a true difference upon ligand binding.

### Chemical analysis of Kvβ2 modification by L-CSNO.

L-CSNO (5 μM) was incubated with equimolar purified Kvβ2 (200 μg/mL ~ 5 μM; MyBioSource) in PBS in the dark at room temperature for 5 minutes, then centrifuged across a 10 kDa ultrafiltration microfuge tube (Thermo Fisher Scientific) (13,000 rpm; 5 minutes; ref. 23). Cold PBS (100 μL) was added to the high-mass fraction, which was spun again for 5 minutes. The low-mass fraction was assayed by copper-cysteine reduction-chemiluminescence (GE-Sievers NOA 280) as previously described (7, 8, 46). The rinsed high-mass fraction was then assayed by reduction-chemiluminescence. Note that the T1 domain of the Kvα protein we studied (MyBioSource) has no cysteines and was not assayed for *S*-nitrosylation.

### Biotin substitution analysis for NO-substituted cysteines in Kv proteins.

CHO cells expressing all 3 Kv proteins were plated on in 6 wells of a 6 well plate. Once confluent, cells were incubated with no treatment, 500 μM L-CSNO, or 500 μM L-CSNO plus 10 mM leucine for 2 minutes. The following steps were performed in the dark. Wells were then washed with cold PBS and lysed in HEN buffer (250 mM HEPES pH 7.7, 1 mM EDTA, 0.1 mM neocuproine). Samples sat on ice for 30 minutes and then centrifuged at 3000*g* to remove cellular debris. After protein assay, concentrations were adjusted to 0.8 μg/μL with HEN buffer and 0.4% CHAPS. Free thiols were blocked with 0.2% *S*-methyl methanethiosulfonate, followed by rotation at 50°C. Proteins were precipitated with acetone and resolved in HENS buffer (250 mM HEPES pH 7.7, 1 mM EDTA, 0.1 mM neocuproine, 1% SDS). Samples were split, and half were incubated with 15 mM ascorbate for 2 minutes at room temperature. All samples were then incubated for 4 hours in the dark with rotation with 50 μL prepared thiopropyl sepharose –b (GE Health Care). After incubation, beads were stringently washed, eluted with tris(2-carboxythyl) phosphine (TCEP), and incubated with trypsin (Promega) in preparation for LC-MS (26).

### Isolation, culture, and electrophysiology of nodose/petrosal ganglia neurons.

Peripheral respiratory control neurons, from CSN to petrosal and nodose ganglia, were excised en bloc from P5–P21 WT and Kv1.1^–/–^ mice,using techniques previously described (27). Tissue was collected after isoflurane anesthesia and decapitation. The ganglia were incubated in Earle’s balanced saline solution (Gibco) containing 40 mg/mL trypsin (Worthington, TLR3), 2 mg/mL cysteine (MilliporeSigma C-7880), 0.5 mM EDTA, and 1.5 mM CaCl_2_, for 30 minutes at 37°C. The enzyme-containing medium was then replaced with 3.3 mL DMEM/F-12 containing 5% FBS, 1% PSN, and 0.5 mg albumin (MilliporeSigma, A-2153). The tissue was triturated to disperse the cells and subsequently placed into 35 mm petri dishes. Electrophysiological experiments were performed on isolated neurons 24–48 hours after plating using the whole-cell patch technique under voltage-clamp conditions. Data were digitized and analyzed using pClamp9 software (Axon Instruments). Series resistance (range, 4–12 MΩ) was 70% compensated. Electrodes (3.0–5.0 MΩ) were prepared from 7052 or 8161 glass (World Precision Instruments). For isolation of potassium currents, the bath solution contained the following (in mM): 140 *N*-methyl-D-glucamine, 5.4 KCl, 1.0 MgCl_2_, 0.02 CaCl_2_, and 10 HEPES, pH adjusted to 7.3 with KOH. The pipette solution contained the following (in mM): 145 K-aspartate, 1.95 CaCl_2_, 2.2 EGTA, 2 MgCl_2_, 10 glucose, and 5 HEPES, pH adjusted to 7.2 with KOH. Total K^+^ currents were elicited by slow voltage ramps (14 mV/100 ms) from –80 to +40 or +60 mV from a holding potential of –60 mV. DTx (Calbiochem; 100 nM) was used to identify Kv1.1, Kv1.2, and Kv1.6 current. L-CSNO, D-CSNO, (10 μM to 1 mM), and DTx were applied from a large-bore perfusion needle placed within 150 μm of the cell body.

### L-CSNO dose-response studies using murine respiratory ganglia.

L-CSNO is labile, with a half-life on the order of 1–10 minutes, degrading on conditions (17, 32). To accurately assess the concentration of L-CSNO at the point of its interaction with tissue in patch clamp studies, levels would need to be measured in a very small volume in situ. There were only about 10 cells on the coverslip/in the dish in the study described above, which contained about 2 mL control solution. The cell was perfused rapidly, first with control and then with test reagent from a pipette placed within at least 100 μm of the cell, so it was flooded with solution continuously until the response stabilized. Therefore, we assessed tissue concentration in situ based on half-life. Stability of L-CSNO in running buffer (as above) and in the presence of murine brain homogenates was assayed by reduction-chemiluminescence as above (46).

### Conscious plethysmography studies.

All studies used adult male Sprague-Dawley rats (10–12 weeks of age) obtained from Harlan. Rats were anesthetized by a bolus injection of pentobarbital sodium (50 mg/kg, i.p.). Rat body temperatures were kept at 37.0°C ± 0.2°C via a rectal probe connected to a thermostat-controlled heating pad (Harvard Apparatus). A catheter (a length of PE-10 melded into ends of PE-50) was inserted into a jugular vein for later infusion of test drugs and at the time of surgery to provide a maintenance infusion of pentobarbital sodium (5 mg/kg). A catheter (PE-50) was inserted into a femoral artery of each rat to record systolic, diastolic, pulsatile, and mean arterial blood pressure (MAP). The left common carotid artery was exposed and separated from the vagus and sympathetic trunks. Two 3.0 silk ligatures (2 cm apart) were placed around the common carotid artery and pulled tight to temporarily occlude blood flow (less than 60 seconds). A small hole was placed in the artery between the ligatures with a 23 gauge needle, and a nonocclusive catheter (PE-10) was inserted with the extruded tip into the trifurcation of the common carotid artery into the external and internal carotid arteries and the occipital artery, such that the tip of the catheter lay within 1–2 mm of the CB artery. Note that the volume of each intra-arterial catheter, for example 109 ± 2 μL for the study represented in Figure 1 and Supplemental Figure 1, was measured before implantation. Upon implantation, the catheter was glued in place with Super Glue (Elmer’s Products Inc.), and ligatures were gradually released until full blood flow was restored. The catheters were exteriorized at the back of the neck, and all wounds were coated liberally with triple antibiotic (neomycin, polymyxin B, bacitracin) ointment (Fougera) and then sutured. The rats were returned to their home cages in a room maintained on a 12-hour light/12-hour dark cycle and were allowed 5 days to recover from surgeries. Food and water were freely available. Details of these surgeries were provided previously (47, 48). All studies were performed in a quiet room with relative humidity of 51% ± 2% and room temperature of 21.3°C ± 0.2°C.

On the day of the experiment, pure (96%–99%), neutral pH solutions of L- and D-CSNO were prepared. Stock solutions were kept on ice and in the dark until ready for use (45). The conscious rats were placed in whole-body plethysmography chambers (PLY3223; Data Sciences International) to record frequency of breathing, TV, and V_E_ as described in detail previously (10, 36, 37). The venous catheter was connected to another port on the ceiling of the plethysmography chambers to allow for gradual infusion of drugs via a rodent infusion pump (PHD 22/2000, Harvard Apparatus). The carotid artery catheter was connected to the internal port of a dual-port swivel assembly in the ceiling of the plethysmography chambers to allow for bolus injection of vehicle, L-CSNO, or D-CSNO via a length of external PE-10 tubing melded into small lengths of PE-50 tubing to connect the tubing to the external port of the swivel and the Hamilton microsyringe (MilliporeSigma). In order to monitor MAP continuously, the femoral artery catheter was connected to the other internal port of the swivel assembly, and one end of a length of PE-50 tubing was connected to the matching external port, while the other end was connected to a pressure transducer connected to a bridge AMP and PowerLab that used LabChart 7.0 (AD Instruments) viewing software to record pulsatile arterial pressure and to determine MAP. After a 60 minute acclimatization period to allow the rats to settle, each carotid artery catheter was loaded with vehicle (saline), L-CSNO, or D-CSNO to completely fill the catheter as per the recorded volume. A small volume, 5 μL, was injected to ensure that the first test injection delivered these solutions. The stock solutions of L- or D-CSNO were diluted to 300 nmol/mL (300 pmol/μL) during the acclimatization period. In order to deliver the 2.5, 5, 10, 25, and 50 nmol/kg doses of L- or D-CSNO (given as a slow bolus over 3 seconds) to a 300 g rat, the volumes to be delivered would be 2.5, 5, 10, 25, and 50 μL, respectively. The volumes given to each rat were modified according to their actual body weight. Equal volumes of vehicle (saline) were given to determine the control responses. All drugs, including l-cysteine, 6-[2-hydroxy-1-methyl-2-nitroso-hydrazino]-*N*-methyl-1-hexanamine (MAMAH NONOate) and 1H-[1,2,4]oxadiazolo[4,3-a]quinoxalin-1-one (ODQ), were obtained from MilliporeSigma. l-Cysteine was dissolved in physiological saline (0.9% NaCl). MAMAH NONOate was dissolved in water and diluted in 0.9% saline. ODQ was dissolved in DMSO and diluted in physiological saline (final concentration of DMSO was 0.1%).

### L-CSNO–specific capacitance-based sensor studies.

We use a sensitive L-CSNO sensor as previously published (33). The tip of insulated carbon fiber electrodes (ALA id CFE-2; NPI Electronics) was coated with a thin layer of polydopamine (33–35). Before passivation, polydopamine is an electrophile, and nucleophiles will covalently bind to it. We functionalized the sensor tip in a solution of polyclonal rabbit anti–L-CSNO antibody in a solution of high-pH (pH 8.2) Tris buffer. When L-CSNO binds to the antibody, it changes the local charge environment of the sensor’s tip, changing the signal. The reference electrode was another insulated carbon fiber coated with polydopamine treated with pH 8.2 Tris buffer, but without antibody. The bath was charged by applying a potential step to the Ag/AgCl pellet, and the response to each electrode was recorded (33). The maximum response current from the sensing electrode is dependent upon the fraction of anti–L-CSNO antibodies binding L-CSNO. This sensor showed a significant change in maximum response in the presence of 4 pM L-CSNO and 40 μM SNO-albumin and albumin, but no significant change in response to other ligands, similar to L-CSNO (SNO-cysteamine, SNO-glutathione, and l-cysteine).

For each experiment, 3 sensing runs were conducted by charging the sensing and reference electrode with a 1 second, +50 mV direct current injection, and allowing the electrodes to discharge for 1 second before charging the electrodes with a –50 mV direct current injection and again allowing them to discharge. The difference between the first 10 ms of the discharge current between when the probe was positively charged and negatively charged served as the signal for the sensor. We then collected 3 sensing experiments with the electrodes in 10 mM Tris-buffered saline, pH 7.4 (running buffer), to serve as a baseline signal. We then perfused in 50 mL running buffer and took an additional 3 readings to assess electrode stability. Finally, we incubated the sensing and reference electrodes in 10 mL running buffer mixed with ligand or biological fluid and allowed it to incubate for 10 minutes. Afterward, the sensor was washed with 50 mL running buffer, and a final 3 experiments were performed to determine the signal we obtained from our solution. Venous or arterial blood was drawn into a heparinized syringe and immediately diluted 1:7 in Tris buffer (above). It was placed in a petri dish and analyzed using the L-CSNO sensor relative to the reference electrode (see above).

### Statistics.

Except as discussed in individual analyses above (for example, in the *Proteomics* section, above), all data are presented as mean ± SEM and were analyzed by 1-way or repeated-measures ANOVA followed by Student’s modified 2-tailed *t* test with Bonferroni’s corrections for multiple comparisons between means. Multiple comparisons were analyzed with Mann-Whitney rank-sum *t* test and Kruskal-Wallis test. A corrected *P* value less than 0.05 was taken to denote statistical significance.

### Study approval.

All protocols involving animals were carried out in accordance with the NIH *Guide for the Care and Use of Laboratory Animals* (49). The protocols were reviewed and approved by the Institutional Animal Care and Use Committee at Case Western Reserve University.

## Author contributions

BG, JB, DK, NM, PG, JNB, and SJL designed the research studies. BG, LS, JB, JS, DK, JK, NM, CAH, PW, PG, STB, TS, and SJL conducted experiments. BG, LS, JB, JS, DK, JK, NM, PW, KM, TSM, STB, TS, SJL, and TL analyzed data. BG, LS, JB, JS, DK, JK, NM, JNB, SJL, and TL prepared and edited the manuscript.

## Supplementary Material

Supplemental data

## Version 1 08/13/2020

In-Press Preview

## Version 2 09/17/2020

Electronic publication
