# Supplementary material for: Voltage-gated potassium channel proteins and stereoselective S-nitroso-l-cysteine signaling
Source: JCI Insight. 2020 Sep 17;5(18):e134174. doi: 10.1172/jci.insight.134174 (PMC7526540; doi:10.1172/jci.insight.134174)
Supplement: Supplemental data [file jciinsight-5-134174-s259.pdf]

Supplementary Materials for

Voltage-gated potassium channel proteins and stereoselective S-nitroso-L-cysteine signaling

Benjamin Gaston[1,2,3], Laura Smith[2], Jürgen Bosch[2], James Seckler[2], Diana Kunze[4], Janna Kiselar[5], Nadzeya Marozkina[2], Craig A. Hodges[2], Patrick Wintrobe[6] , Kellen McGee[2], Tatiana S. Morozkina[7], Spencer T. Burton[2], Timothy Strassmaier[8], James N. Bates[9], Stephen J. Lewis[2,10*]

Affiliations:

[1]Riley Hospital for Children.

[2] Case Western Reserve University Department of Pediatric Pulmonology.

[3] Case Western Reserve University Department of Physiology and Biophysics.

[4] Case Western Reserve University Metro Health System.

[5]Case Western Reserve University Department of Proteomics and Bioinformatics.

[6]University of Maryland Department of Pharmaceutical Sciences.

[7]Belarussian State Medical University (posthumous).

[8]Nanion Inc

[9]University of Iowa Department of Anesthesia.

[10]Case Western Reserve University Department of Pharmacology.

Supplemental Figures

Supplemental Figure S1

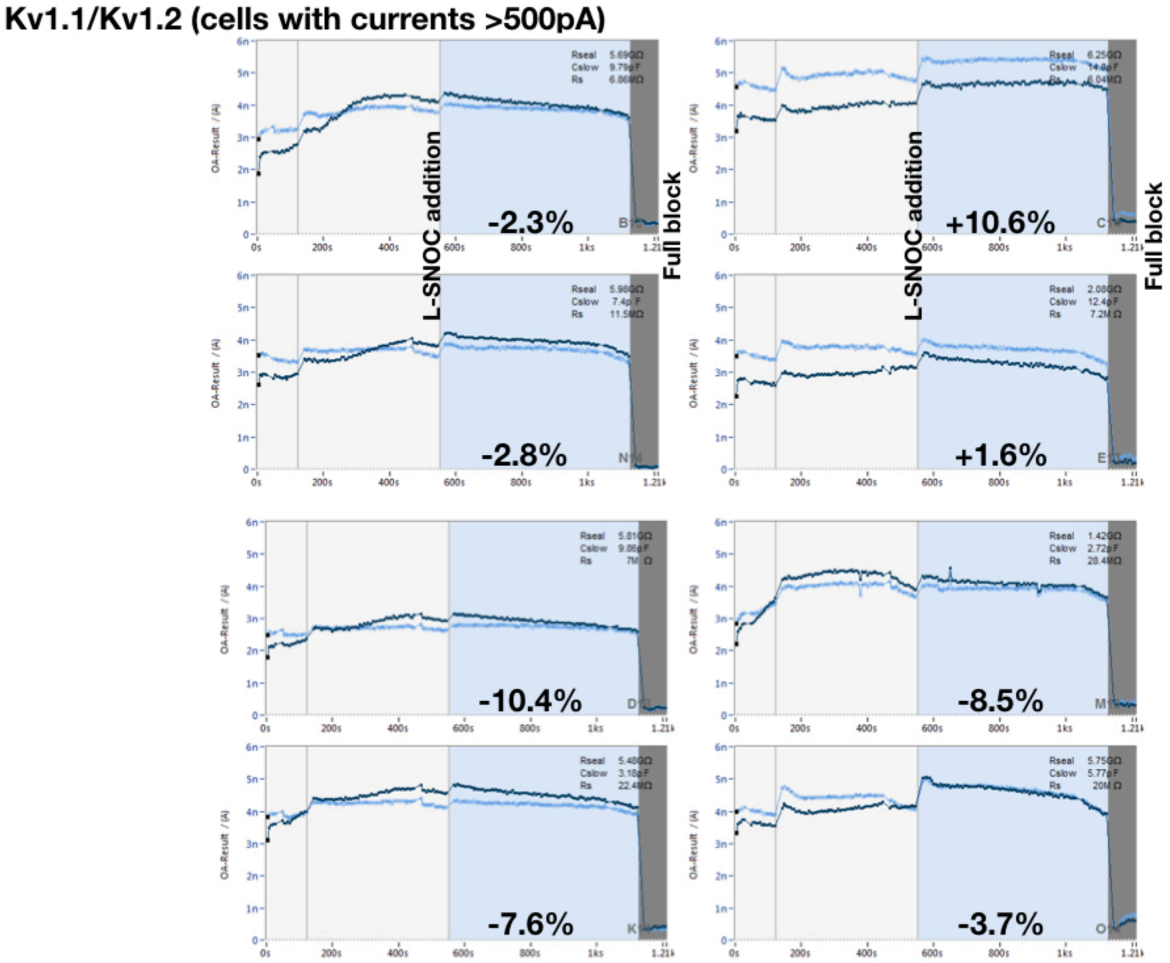

Fig. S1. Traces of individual cells used for generation of Fig. 2g. Eight representative wells that passed the quality criteria described in the materials & methods section for the Syncropatch are shown for either the Kv1.1/Kv1.2 or Kv1.1/Kv1.2/Kvβ2 cell line. After cell capture and seal enhancement, the cells are allowed to rest for 500 s. Addition of L-CSNO is indicated by the blue shaded area and allowed to incubate for approximately 500 s prior to the addition of the full block (20 mM TEA+10 mM 4-AP) in dark grey. Conductance of the first trace (blue) and last trace (black) is shown in each panel. The percentage of block compared to the full block is indicated in each panel. The mean block for Kv1.1/Kv1.2 is -2.9% while for Kv1.1/Kv1.2/Kvβ2 it is -23.1%.

Supplemental Figure S2

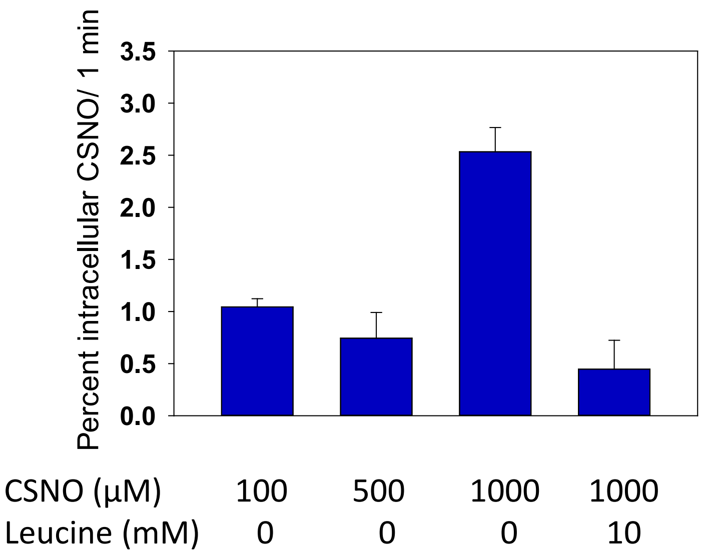

Fig. S2. Fraction of L-CSNO entering the cell in one minute. CHO cells overexpressing Kv1.1, Kv1.2 and Kvβ2 were grown on coverslips till 90% confluence; 24 hours before experiment cell media was replaced with serum-free media. CHO cells were treated (n = 3 each) with 100 µM CSNO, 500 µM CSNO, 1000 µM CSNO, and 1000 µM CSNO with 10 mM leucine (to inhibit the LAT transporter [26]) for 1 min. At time 0, 100 µl of media from each sample was collected and frozen in dry ice in the dark. At 1 min, cells rapidly washed 3x in PBS, pipetted dry, scraped, and frozen in the dark in dry ice. Baseline supernatant and 1 minute cell lysate were assayed using copper-cysteine chemiluminescence assay (as described previously, *42*). Inside/ outside CSNO ratio was calculated for each condition. Leucine significantly inhibited L-CSNO uptake ($p < 0.05$).

Supplemental Figure S3

a.

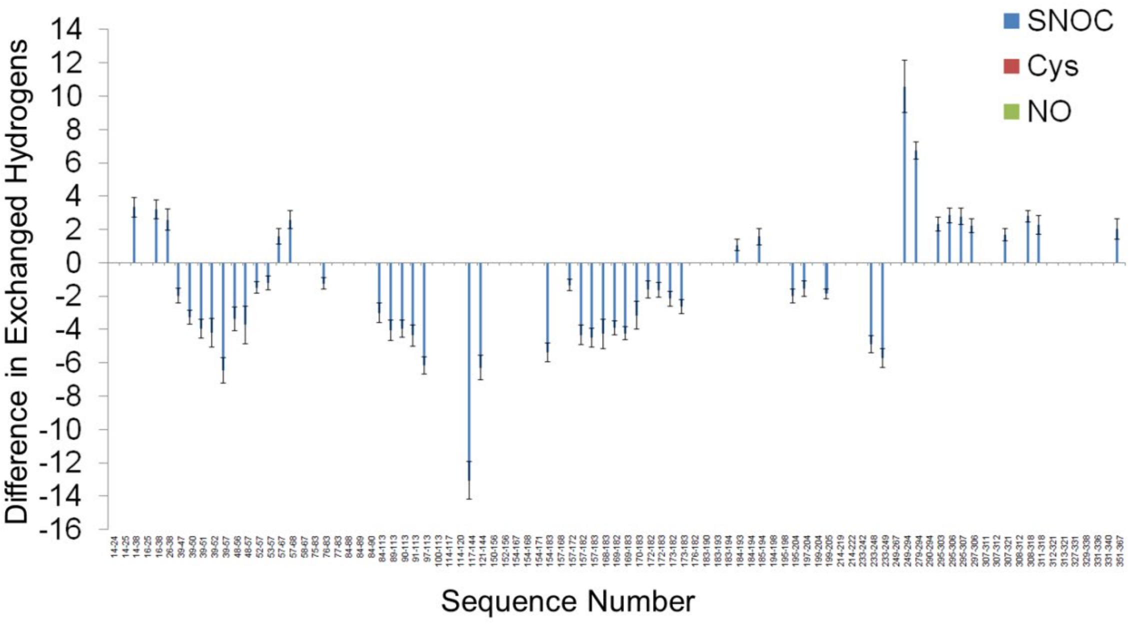

b.

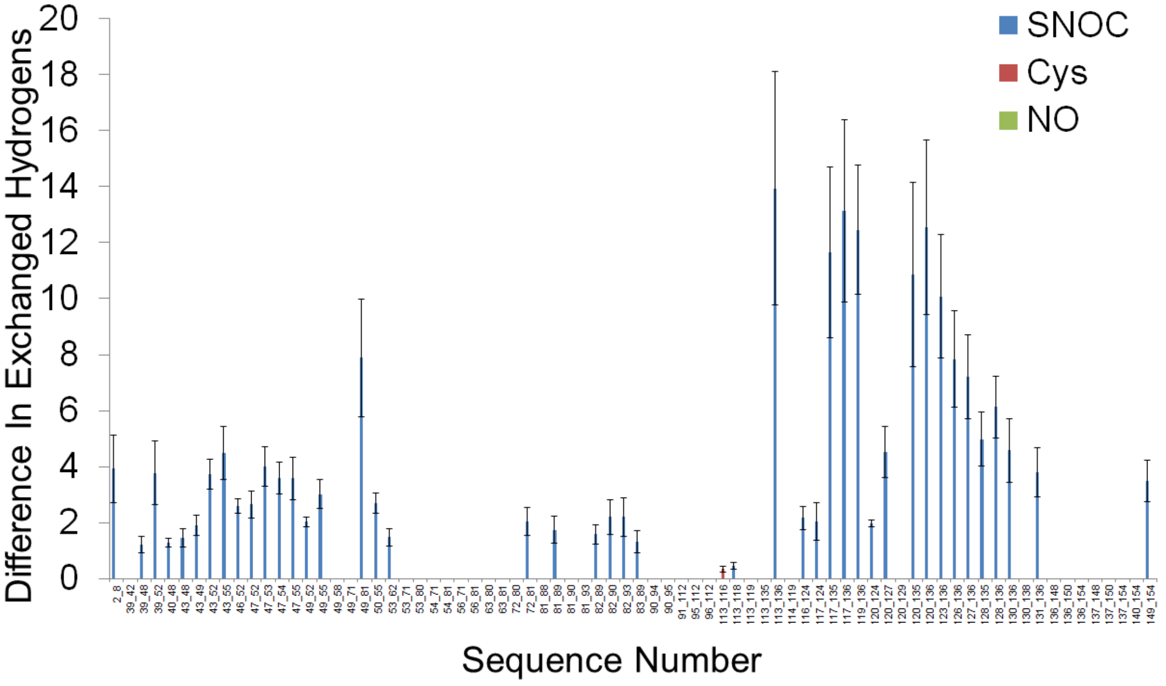

Fig. S3. Hydrogen-deuterium exchange data peptide analyses for Kv α 1.1 T1 and Kvβ2. (a). Peptide analysis of the Kv α 1.1 T1 data. The difference in number of deuteria exchanged in individual peptic fragments between unliganded Kv α 1.1 T1 domain and Kv α 1.1 T1 domain bound to either: SNO-Cysteine (Blue), Cysteine (Red), or Ethyl Nitrite (Green). Only peptic fragments showing a significant difference are shown. Kv α 1.1 T1 domain bound to Ethyl Nitrite showed no significant change in deuterium exchange strongly suggesting that the presence of this ligand causes no significant change in structure or dynamics. A single peptic fragment showed a slight, but significant stabilization in the presence of cysteine, suggesting that cysteine causes slight stabilization where it binds, but has no global effect on dynamics or structure. (**b**). ***Peptide analysis of HDX Kvβ2 data***. The difference in number of deuteria exchanged in individual peptic fragments between unliganded Kvβ2 and Kvβ2 bound to either: SNO-Cysteine (Blue), Cysteine (Red), or Ethyl Nitrite (Green). Only peptic fragments showing a significant difference are shown. Kvβ2 bound to Cysteine and Ethyl Nitrite showed no significant change in deuterium exchange strongly suggesting that the presence of these ligands causes no significant change in structure or dynamics.

Supplemental Figure S4

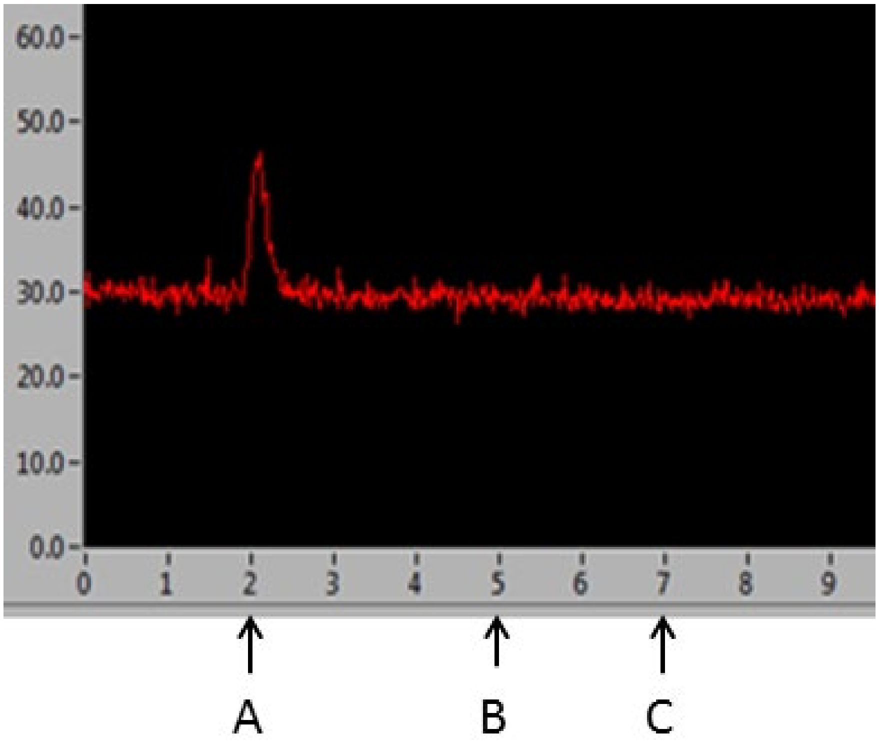

Fig. S4. Chemical Analysis of Kvβ2 modification by L-CSNO. L-CSNO (5 µM) was incubated with equimolar purified Kvβ2 (200 µg/mL ~ 5 µM; MyBioSource, San Diego, CA) in PBS in the dark at room temperate for 5 min, then centrifuged across a 10 kDa ultrafiltration microfuge tube (Fisher) (13,000 RPM; 5 min). 100 µL of cold PBS was added to the high mass fraction, which was spun again for 5 min. A low mass fraction (A) was assayed by copper-cysteine reduction-chemiluminescence (*31,42*). The rinsed high mass fraction was then assayed by reduction-chemiluminescence (n = 2; B and C). The experiment was performed on two aliquots each of Kvβ2 on two separate days (n = 4 total). No S-nitrosylation of Kvβ2 was observed. Note that the T1 domain of the Kvα protein we studied (MyBioSource, San Diego, CA) has no cysteines and was not assayed for S-nitrosylation.

Figures S5-S7. The injections of L-CSNO (labeled SNO-L-CYS; 2.5 to 50 nmol/kg, IA) elicited dose-dependent increases in frequency and tidal volume (Fig. S5, Panels A and B, respectively) and therefore minute ventilation (Fig. 5, Panel C) of between 130 to 180 sec in duration. The injections of SNO-D-CYS elicited substantially smaller ventilatory responses (Fig. S5, Panels A and B; Fig. 5, Panel C). These injections ofL-CSNO elicited relatively minor changes in MAP with only the 50 nmol/kg dose producing a significant hypotensive response (Fig. S5, Panel C). The injections of D-CSNO (labeled SNO-D-CYS) did not lower MAP at any dose (Fig. S5, Panel C). The magnitudes and durations of the ventilatory and hypotensive responses elicited by the 25 nmol/kg injection of L-CSNO are summarized in Fig. 5 (Panel D). These data demonstrate that the ability of L-CSNO to increase minute ventilation is not related to its propensity to lower MAP. The relatively minor falls in MAP elicited by the 25 and 50 nmol/kg intra-arterial doses of SNO-L-CYS are similar to those reported following intravenous (jugular vein) injections of these doses in conscious rats noting that higher doses of L-CSNO elicit substantially greater hypotensive responses. The injections of vehicle elicited minor responses that were not significant at any volume. For example the highest injection volumes (51.7 ± 0.4 μL, those which were used to deliver the 50 nmol/kg doses of L-CSNO or D-CSNO) elicited changes in frequency of breathing, tidal volume, minute ventilation and MAP of +0.5 ± 1.1%, +1.3 ± 0.8 %, +1.7 ± 1.0 %, and +0.7 ± 0.6 %, respectively ($P > 0.05$, for all responses).

Effects of intravenous infusions of vehicle, ODQ or L-SMC on the changes in minute ventilation and MAP elicited by bolus intra-carotid artery injections of L-CSNO. As shown in Table S3, resting parameters before commencing the above infusions were similar to one another (P = NS for all comparisons). Infusion of vehicle did not alter any baseline value (P = NS for all comparisons). Infusion of ODQ elicited minor but significant ($P < 0.05$) increases in frequency of breathing and minute ventilation that were sustained throughout the infusion period whereas it did not alter tidal volume or MAP. Infusion of L-SMC elicited minor but significant decreases in frequency of breathing, tidal volume and minute ventilation ($P < 0.05$) and a somewhat more substantial increase in MAP ($P < 0.05$), responses that were all sustained throughout the infusion period. Similar to findings described above, slow bolus injections of SNO-L-CYS elicited dose-dependent increases in frequency of breathing, tidal volume and minute ventilation whereas they elicited dose-dependent decreases in MAP. Changes in frequency of breathing, tidal volume and MAP elicited by L-CSNO (Fig. S6, Panels A, B and C respectively) and minute ventilation (Fig. 5, Panel E) were not affected by ODQ. In contrast, the L-CSNO responses were markedly attenuated in rats receiving infusion of L-SMC (Fig.5, Fig. S6).

Slow bolus injections of the nitric oxide donor, MAHMA NONOate, elicited minor decreases in frequency of breathing but no changes in tidal volume, which resulted in minor reductions in minute

ventilation (Fig. S7, panels A, B and C, respectively). These injections of MAHMA NONOate elicited dose-dependent and pronounced reductions in MAP. The minor changes in frequency of breathing and minute ventilation and the decreases in MAP elicited by MAHMA NONOate were markedly diminished in rats receiving the infusion of ODQ (Fig. S7, panels A to D). The minor MAHMA NONOate-induced decreases in frequency and minute ventilation were apparently diminished in the rats receiving the infusion of L-SMC (Fig. S7, panels A and C, respectively). However, this is most likely because L-SMC itself elicited minor reductions in these parameters (Table S3) thereby precluding any further falls in these parameters. The observation that L-SMC did not affect the dose-dependent falls in MAP elicited by MAHMA NONOate (Fig. S7, Panel D) certainly suggests that L-SMC does not interfere with the biological actions of nitric oxide.

Supplemental Figure S5

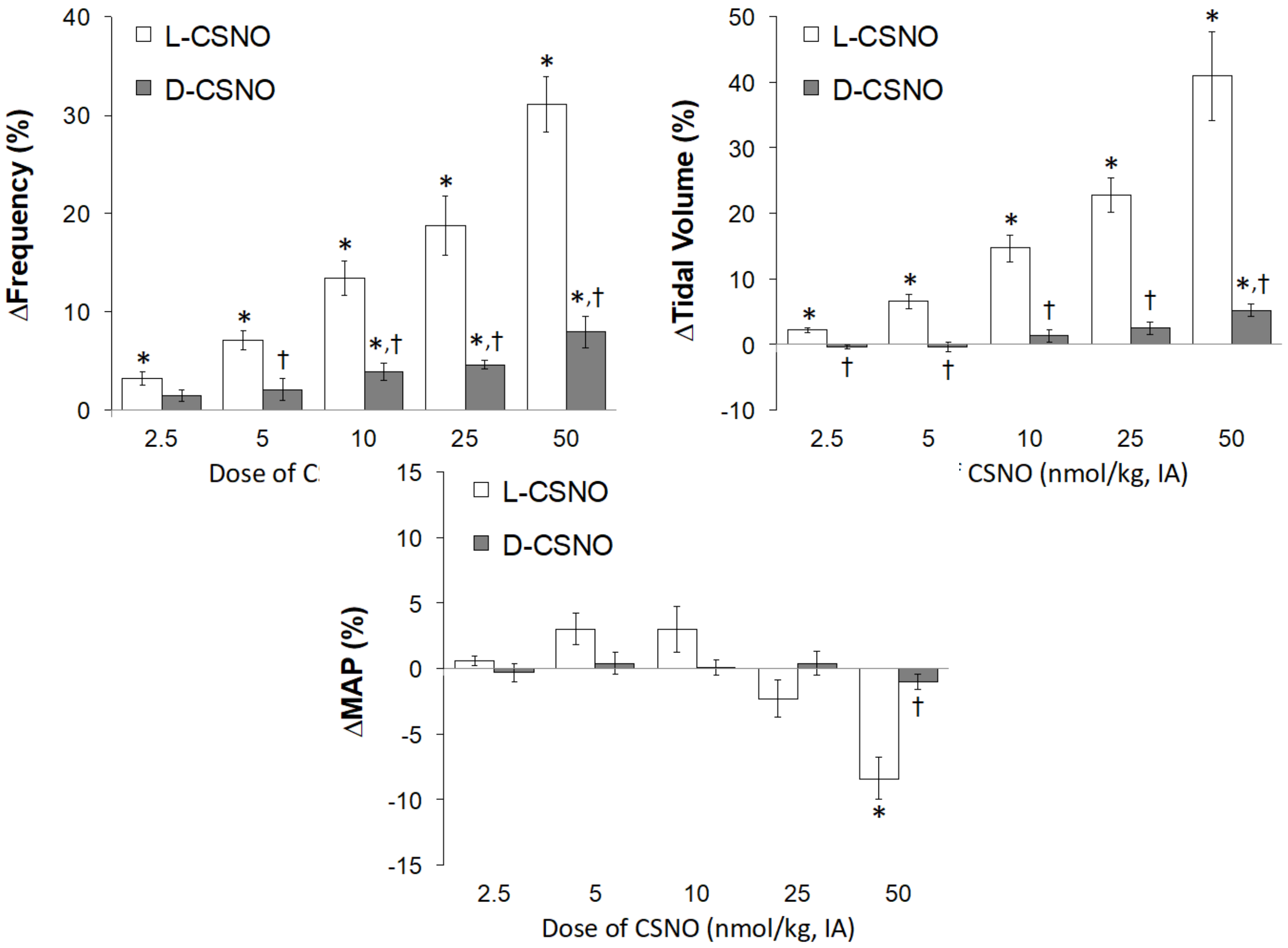

Fig. S5. ***L-CSNO-induced responses are greater than those elicited by D-CSNO.*** Maximal changes in frequency of breathing **(A)**, tidal volume **(B)**, and mean arterial blood pressure (MAP) **(C)**, elicited by arterial injections of L-CSNO or D-CSNO in conscious Sprague-Dawley rats (n=9). The data are presented as mean ± SEM. *P < 0.05, significant response. †P < 0.05, D-CSNO *versus* L-CSNO.

Supplemental Figure S6

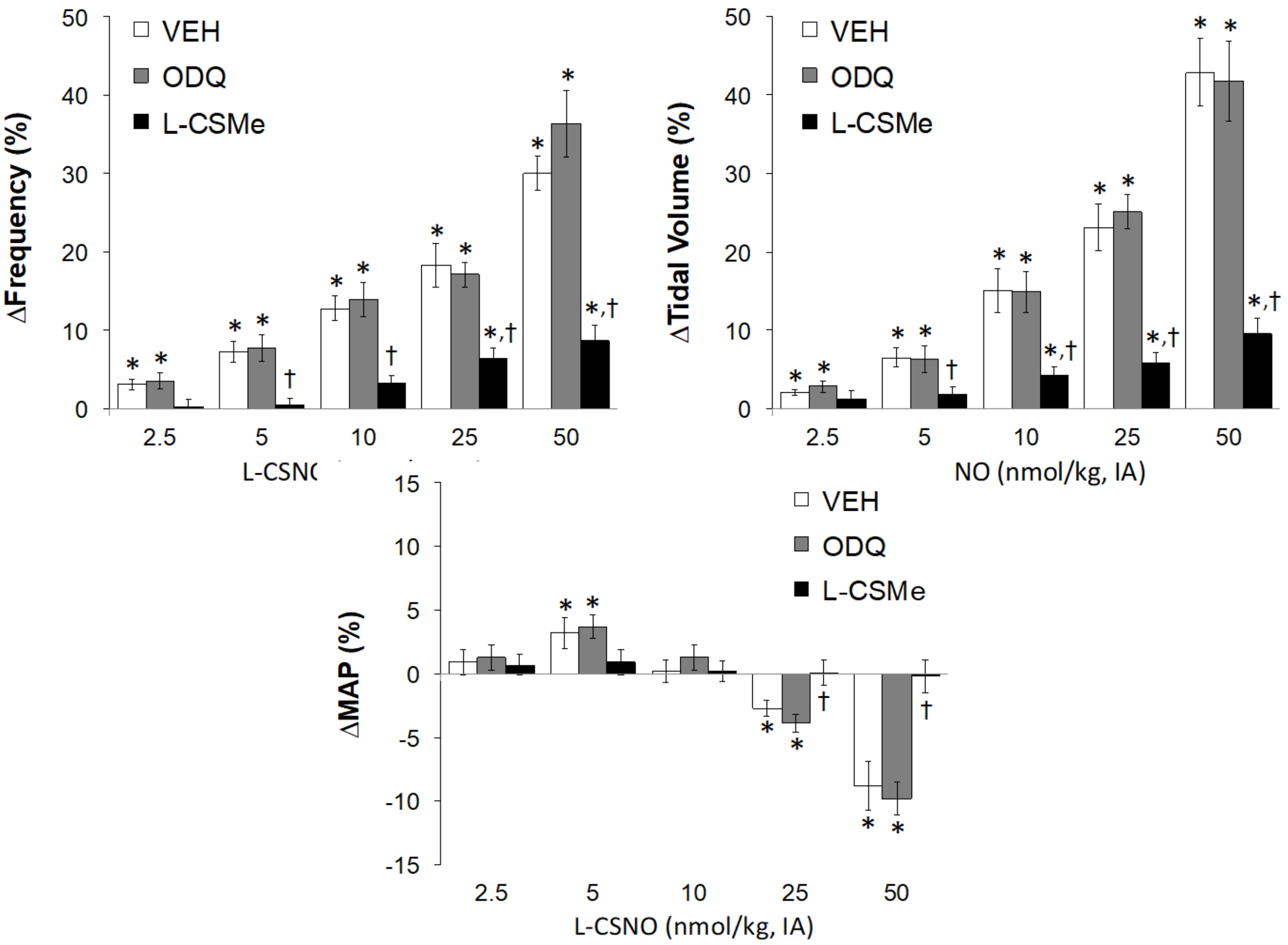

Fig. S6. L-CSMe blocks the ventilatory responses elicited by L-CSNO. In the infusion studies, the maximum changes in frequency of breathing, tidal volume, minute ventilation and MAP elicited by slow bolus (3 s) intra-carotid artery injections of L-CSNO were determined in separate groups of conscious Sprague-Dawley rats receiving intravenous infusions of vehicle (0.1% DMSO in saline, 20 µL/min), ODQ (2 mg/kg bolus followed by 50 µg/kg/min) or L-CSMe (10 µmol/kg/min). The infusions were commenced 30 min prior to the first bolus injection of vehicle, L-CSNO or the nitric oxide donor, MAHMA NONOate, and continued for another 90 min to allow the injections to be given. As such the

rats that received infusion of vehicle (n = 9), ODQ (n = 9) or L-CSMe (n = 9) subsequently received bolus intra-arterial injections of vehicle, L-CSNO and MAHMA NONOate. In 3 rats, the order of injections was vehicle, (5 injections of ascending volume), MAHMA NONOate (2.5, 5, 10, 25 and 50 nmol/kg) and then L-CSNO (2.5, 5, 10, 25 and 50 nmol/kg), in another 3 rats the order was MAHMA NONOate, vehicle and then L-CSNO (volumes/doses as above), and in the other 3 rats the order was L-CSNO, vehicle and then MAHMA NONOate (volumes/doses as above). It required about 90 min to complete the injection protocols including time needed to replace the injection solutions. As such the rats received approximately 2.4 mL over a 2 hour period in total.

Supplemental Figure S7

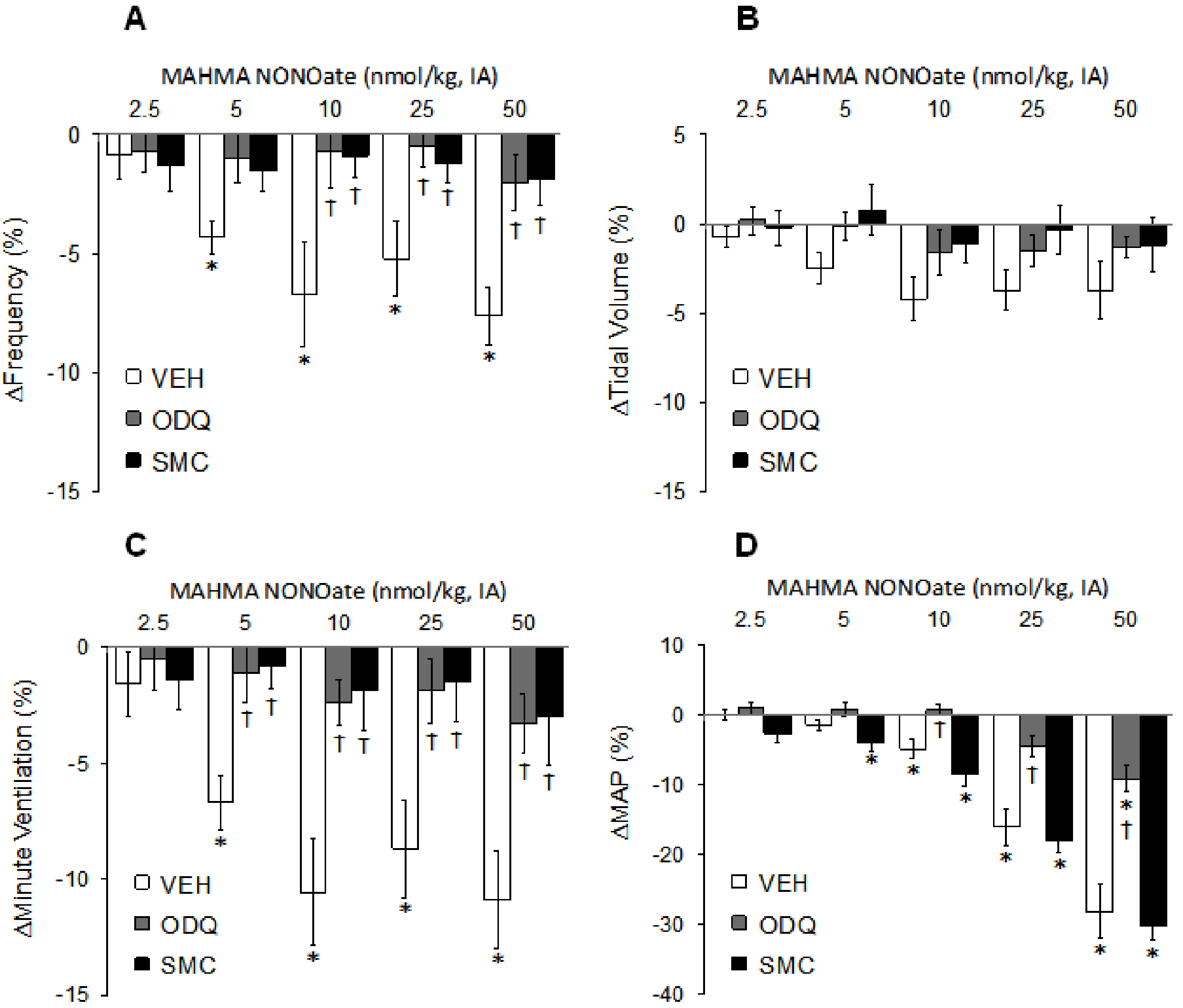

Fig. S7. NO-mediated reductions in minute ventilation and MAP are blocked by ODQ. Maximal changes in frequency of breathing **(A)**, tidal volume **(B)**, minute ventilation **(C)** and mean arterial blood pressure (MAP) **(D)**, elicited by bolus intra-carotid artery injections of MAHMA NONOate in Sprague-Dawley rats receiving intravenous infusions of vehicle (0.1% DMSO in saline, 20 µL/min), ODQ (2 mg/kg bolus followed by 50 µg/kg/min) or L-SMC (1 µmol/kg/min). There were 9 rats in each group. The data are presented as mean ± SEM. *$P < 0.05$, significant response. †$P < 0.05$, SMC *versus* VEH and ODQ.

Supplemental Figure S8

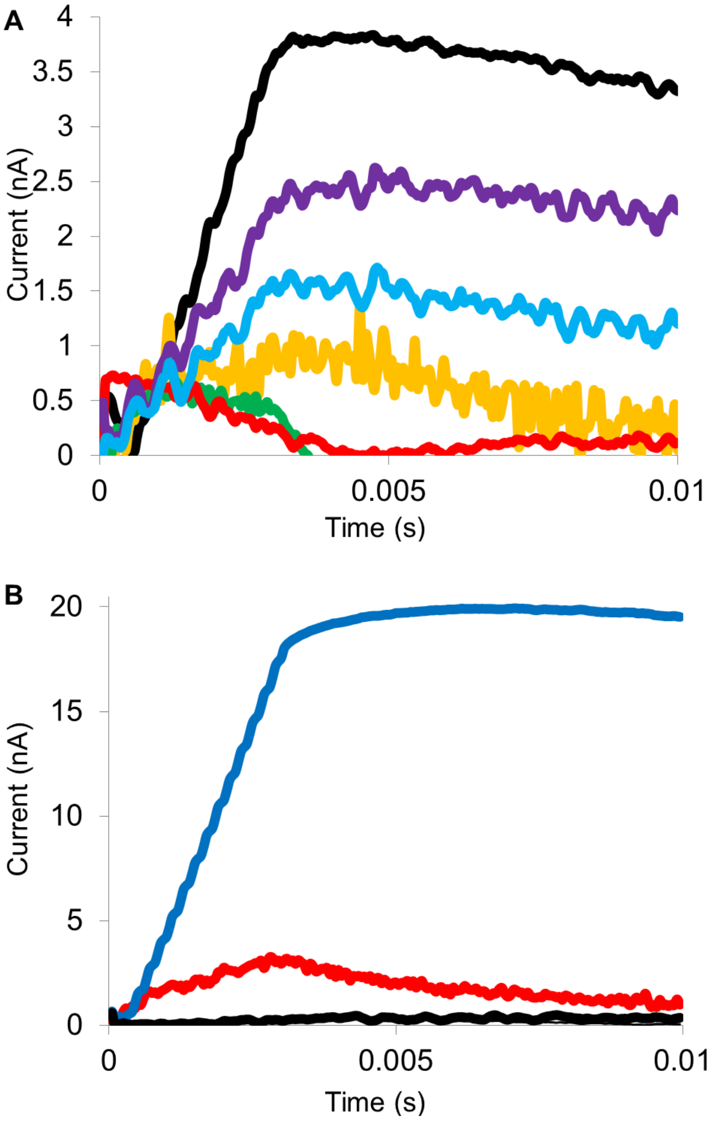

Fig. S8. Assays for S-nitrosocysteine in whole blood. **Panel A:** Current traces of the sensor after 10 minutes of incubation in either: 4 pM L-CSNO (Black), 4 pM S-nitrosocysteamine (Orange), 4 nM GSNO (Green), 4 nM L-cysteine (Red), 40 µM S-nitroso-albumin (Purple), 40 µM Albumin (Blue; note that this is consistent with previous data suggesting endogenous S-nitrosylation of S-nitroso-albumin. Note that sensitivity for all endogenous S-nitrosothiols except S-nitrosocysteamine is three log orders lower than that L-CSNO. **Panel B:** Current traces of the sensor after 10 minutes of incubation in either: Venous Blood (Blue), Arterial Blood (Red), or Tris (Black).

Supplemental tables

Table S1. Data from the native PAGE with/without inhibitors

Bold text: classes of proteins

Italics: *Protein classes identified both by native page and (Table S3) affinity chromatography.*

Bold italics underlined: Specific proteins of interest in this manuscript, including MASCOT score and number of unique peptides.

**1. Na/K ATPases**	
AT1A1_MOUSE	Sodium/potassium-transporting ATPase subunit alpha-1
AT1A2_MOUSE	Sodium/potassium-transporting ATPase subunit alpha-2
AT1A3_MOUSE	Sodium/potassium-transporting ATPase subunit alpha-3
AT1B1_MOUSE	Sodium/potassium-transporting ATPase subunit beta-1
AT1B2_MOUSE	Sodium/potassium-transporting ATPase subunit beta-2
**2. Ca ATPase**	
AT2B2_MOUSE	Plasma membrane calcium-transporting ATPase 2
**3. Voltage gated Ca channels.**	
CA2D1_MOUSE	Voltage-dependent calcium channel subunit alpha-2/delta-1
**a. R-type**	
CAC1E_MOUSE	Voltage-dependent R-type calcium channel subunit alpha-1E
**b. L-type**	
CACB1_MOUSE	Voltage-dependent L-type calcium channel subunit beta-1
CACB4_MOUSE	Voltage-dependent L-type calcium channel subunit beta-4
**4. Carbonic anhydrases (1 and 4)**	
CAH1_MOUSE	Carbonic anhydrase 1
CAH4_MOUSE	Carbonic anhydrase 4
**5. CAM kinase-like vesicle-associated**	
CAMKV_MOUSE	CaM kinase-like vesicle-associated protein
**6. Glutamate decarboxylase**	
DCE1_MOUSE	Glutamate decarboxylase 1
**7. Excitatory amino acid transporters (1 and 2)**	
EAA1_MOUSE	Excitatory amino acid transporter 1
EAA2_MOUSE	Excitatory amino acid transporter 2
**8. GABA B receptor subunits**	
GABR1_MOUSE	Gamma-aminobutyric acid type B receptor subunit 1
GBRB2_MOUSE	Gamma-aminobutyric acid receptor subunit beta-2
**9. G protein subunits**	
GBB1_MOUSE	Guanine nucleotide-binding protein G(I)/G(S)/G(T) subunit beta-1
GBB2_MOUSE	Guanine nucleotide-binding protein G(I)/G(S)/G(T) subunit beta-2
GBG12_MOUSE	Guanine nucleotide-binding protein G(I)/G(S)/G(O) subunit gamma-12
GBG7_MOUSE	Guanine nucleotide-binding protein G(I)/G(S)/G(O) subunit gamma-7
GNA11_MOUSE	Guanine nucleotide-binding protein subunit alpha-11
GNAI2_MOUSE	Guanine nucleotide-binding protein G(i) subunit alpha-2
GNAL_MOUSE	Guanine nucleotide-binding protein G(olf) subunit alpha
GNAO_MOUSE	Guanine nucleotide-binding protein G(o) subunit alpha

GNAS1_MOUSE Guanine nucleotide-binding protein G(s) subunit alpha isoforms XL

10. Soluble G cyclase

GCYB1_MOUSE Guanylate cyclase soluble subunit beta-1

11. Orphan GPCR

GP158_MOUSE Probable G-protein coupled receptor 158

12. Neural G-protein regulated protein.

GRIN1_MOUSE G protein-regulated inducer of neurite outgrowth 1

13. Glutamate receptors

GRIA2_MOUSE Glutamate receptor 2
GRM1_MOUSE Metabotropic glutamate receptor 1
GRM2_MOUSE Metabotropic glutamate receptor 2
GRM3_MOUSE Metabotropic glutamate receptor 3

14. ATP-sensitive I-K channel

IRK10_MOUSE ATP-sensitive inward rectifier potassium channel 10

15. IP3 receptor

ITPR1_MOUSE Inositol 1,4,5-trisphosphate receptor type 1

16. Ca-activated K channel.

KCMA1_MOUSE Calcium-activated potassium channel subunit alpha-1

17. Voltage-gated K channels.

a. Subunit A.

KCNA1_MOUSE **Potassium voltage-gated channel subfamily A member 1 (MASCOT score 490; 2 unique)**

KCNA2_MOUSE ***Potassium voltage-gated channel subfamily A member 2 (MASCOT score 406, 1 unique)***

KCNA6_MOUSE ***Potassium voltage-gated channel subfamily A member 6 (MASCOT score 213, 1 unique)***

b. Subunit B.

KCNAB2_MOUSE ***Voltage-gated potassium channel subunit beta-2 (MASCOT score 52, 1 unique)***

c. Sub-family KQT member 2

KCNQ2_MOUSE ***Potassium voltage-gated channel subfamily KQT member 2***

18. Magnesium transporter

MMGT1_MOUSE Membrane magnesium transporter 1

19. Na/Ca exchanger

NAC1_MOUSE Sodium/calcium exchanger 1

20. Neurobeachin

NBEA_MOUSE Neurobeachin

21. Neurofascin

NFASC_MOUSE Neurofascin
NLGN2_MOUSE Neuroligin-2

23. Neuroplastin

NPTN_MOUSE Neuroplastin

24. PDE 1B

PDE1B_MOUSE Calcium/calmodulin-dependent 3',5'-cyclic nucleotide phosphodiesterase 1B

25. cGMP PDE

PDE2A_MOUSE cGMP-dependent 3',5'-cyclic phosphodiesterase
PDE1B_MOUSE Calcium/calmodulin-dependent 3',5'-cyclic nucleotide phosphodiesterase 1B

**26. Cyclic nucleotide channel**	
PEX5R_MOUSE	PEX5-related protein
vPDE2A_MOUSE	cGMP-dependent 3',5'-cyclic phosphodiesterase
**27. Renin receptor**	
RENR_MOUSE	Renin receptor
**28. GTPase inhibitor of G protein**	
RGS7_MOUSE	Regulator of G-protein signaling
**29. Sodium and chloride-dependent GABA transporter**	
SC6A1_MOUSE	Sodium- and chloride-dependent GABA transporter 1
**30. Sodium channel**	
SCN9A_MOUSE	Sodium channel protein type 9 subunit alpha
31. Syntaxin binding protein 1	
A, B STXB1_MOUSE	***Syntaxin-binding protein 1***
**32. Enigmatic transmembrane proteins**	
TM111_MOUSE	Transmembrane protein 111
TMM33_MOUSE	Transmembrane protein 33
TMM35_MOUSE	Transmembrane protein 35
**33. TRPV2**	
TRPV2_MOUSE	Transient receptor potential cation channel subfamily V member 2
34. VATPases	
VATL_MOUSE	V-type proton ATPase 16 kDa proteolipid subunit
VA0D1_MOUSE	V-type proton ATPase subunit d 1
VAS1_MOUSE	V-type proton ATPase subunit S1
VATA_MOUSE	V-type proton ATPase catalytic subunit A
VATB2_MOUSE	V-type proton ATPase subunit B, brain isoform
VATC1_MOUSE	V-type proton ATPase subunit C 1
VATD_MOUSE	V-type proton ATPase subunit D
VATE1_MOUSE	V-type proton ATPase subunit E 1
VATG2_MOUSE	V-type proton ATPase subunit G 2
VATH_MOUSE	V-type proton ATPase subunit H
VPP1_MOUSE	V-type proton ATPase 116 kDa subunit a isoform 1
**35. Voltage-dependent anion-selective channel (1, 2 and 3)**	
VDAC1_MOUSE	Voltage-dependent anion-selective channel protein 1
VDAC2_MOUSE	Voltage-dependent anion-selective channel protein 2
VDAC3_MOUSE	Voltage-dependent anion-selective channel protein 3

Table S2. Affinity column results

<u>Italics/Bold/Underlined:</u> Protein of interest to this study (with MASCOT score and number of unique peptides)

Activated RNA polymerase II transcriptional coactivator p15
ADP-ribosylation factor 1 and ADP-ribosylation factor 5
Apolipoprotein O
Calcium/calmodulin-dependent protein kinase type II subunit alpha 2
CB1 cannabinoid receptor-interacting protein 1
Cell division control protein 42 homolog
Cerebellin-1
Cofilin-1
Cysteine and glycine-rich protein 1
Cytochrome c oxidase subunit 4 isoform 1, mitochondrial
Cytochrome c oxidase subunit 5A, mitochondrial
Destrin OS=Mus musculus
Elongation factor 1-alpha 1 and Elongation factor 1-alpha 2
Eukaryotic translation initiation factor 5A-1
Glutathione S-transferase P 1
Golgi-associated plant pathogenesis-related protein 1
Guanine nucleotide-binding protein subunit beta-2-like 1
Hemoglobin subunit alpha and Hemoglobin subunit beta-1
Integrin beta-1-binding protein 1
Interleukin enhancer-binding factor 2
Malignant T cell-amplified sequence 1
NADH dehydrogenase [ubiquinone] 1 alpha subcomplex subunit 13
NADH dehydrogenase [ubiquinone] 1 beta subcomplex subunit 4
NADH dehydrogenase [ubiquinone] iron-sulfur protein 7, mitochondrial
NADH dehydrogenase [ubiquinone] iron-sulfur protein 8, mitochondrial
Peptidyl-prolyl cis-trans isomerase NIMA-interacting 1
Phospholipid hydroperoxide glutathione peroxidase, mitochondrial
Ras-related C3 botulinum toxin substrate 1
Rho-related GTP-binding protein RhoB
Signal recognition particle 14 kDa protein
Syntaxin-binding protein 1
Trafficking protein particle complex subunit 3
Transcription factor A, mitochondrial
Transmembrane protein 65
Uncharacterized protein C6orf125 homolog
<u>Voltage-gated potassium channel subunit beta-2 (Mascot score 196, 2 unique)</u>
Voltage-gated potassium channel B2 Shab subfamily
V-type proton ATPase subunit G 2

Table S3. Resting parameters and effects of infusions

Infusion	Parameter	Pre	Post	%Change
**Vehicle**	n = 9 rats, 81 ± 1 days; 317 ± 2 grams			
	Frequency (breaths/min)	105 ± 3	106 ± 3	+1.3 ± 0.7
	Tidal Volume (ml)	2.31 ± 0.04	2.30 ± 0.05	-0.2 ± 0.5
	Minute Ventilation (ml/min)	242 ± 7	244 ± 7	+1.1 ± 0.6
	MAP (mmHg)	108 ± 2	109 ± 2	+0.9 ± 1.0
**ODQ**	n = 9 rats, 81 ± 1 days; 316 ± 2 grams			
	Frequency (breaths/min)	108 ± 4	111 ± 14	+3.8 ± 1.3*
	Tidal Volume (ml)	2.34 ± 0.06	2.37 ± 0.07	+1.3 ± 0.9
	Minute Ventilation (ml/min)	251 ± 10	263 ± 9	+5.1 ± 1.6*
	MAP (mmHg)	109 ± 2	112 ± 3	+2.8 ± 1.6
**L-SMC**	n = 9 rats, 81 ± 1 days; 317 ± 2 grams			
	Frequency (breaths/min)	104 ± 3	97 ± 2	-6.8 ± 1.0*
	Tidal Volume (ml)	2.35 ± 0.06	2.23 ± 0.03	-4.9 ± 1.3*
	Minute Ventilation (ml/min)	245 ± 11	216 ± 6	-11.4 ± 1.8*
	MAP (mmHg)	109 ± 2	125 ± 3	+15.2 ± 1.8*

MAP, mean arterial blood pressure. ODQ, 1H-[1,2,4]oxadiazolo[4,3,-a] quinoxalin-1-one. SMC, L-S-methylcysteine.The data are presented as mean ± SEM. There were 9 rats in each group. *P < 0.05, significant change compared to vehicle.
